# Mitogenomic architecture and evolution of the soil ciliates *Colpoda*

**DOI:** 10.1128/msystems.01161-23

**Published:** 2024-01-23

**Authors:** Yuanyuan Zhang, Haichao Li, Yaohai Wang, Mu Nie, Kexin Zhang, Jiao Pan, Yu Zhang, Zhiqiang Ye, Rebecca A. Zufall, Michael Lynch, Hongan Long

**Affiliations:** 1 Key Laboratory of Evolution and Marine Biodiversity (Ministry of Education), Institute of Evolution and Marine Biodiversity, KLMME, Ocean University of China, Qingdao, Shandong Province, China; 2 Laboratory for Marine Biology and Biotechnology, Laoshan Laboratory, Qingdao, Shandong Province, China; 3 School of Mathematics Science, Ocean University of China, Qingdao, Shandong Province, China; 4 School of Life Sciences, Central China Normal University, Wuhan, Hubei Province, China; 5 Department of Biology and Biochemistry, University of Houston, Houston, Texas, USA; 6 Biodesign Center for Mechanisms of Evolution, Arizona State University, Tempe, Arizona, USA; University of California, San Diego, California, USA

**Keywords:** ciliated protozoa, mitochondria, evolutionary genomics

## Abstract

*Colpoda* are cosmopolitan unicellular eukaryotes primarily inhabiting soil and benefiting plant growth, but they remain one of the least understood taxa in genetics and genomics within the realm of ciliated protozoa. Here, we investigate the architecture of *de novo* assembled mitogenomes of six *Colpoda* species, using long-read sequencing and involving 36 newly isolated natural strains in total. The mitogenome sizes span from 43 to 63 kbp and typically contain 28**–**33 protein-coding genes. They possess a linear structure with variable telomeres and central repeats, with one *Colpoda elliotti* strain isolated from Tibet harboring the longest telomeres among all studied ciliates. Phylogenomic analyses reveal that *Colpoda* species started to diverge more than 326 million years ago, eventually evolving into two distinct groups. Collinearity analyses also reveal significant genomic divergences and a lack of long collinear blocks. One of the most notable features is the exceptionally high level of gene rearrangements between mitochondrial genomes of different *Colpoda* species, dominated by gene loss events. Population-level mitogenomic analysis on natural strains also demonstrates high sequence divergence, regardless of geographic distance, but the gene order remains highly conserved within species, offering a new species identification criterion for *Colpoda* species. Furthermore, we identified underlying heteroplasmic sites in the majority of strains of three *Colpoda* species, albeit without a discernible recombination signal to account for this heteroplasmy. This comprehensive study systematically unveils the mitogenomic structure and evolution of these ancient and ecologically significant *Colpoda* ciliates, thus laying the groundwork for a deeper understanding of the evolution of unicellular eukaryotes.

**IMPORTANCE:**

*Colpoda*, one of the most widespread ciliated protozoa in soil, are poorly understood in regard to their genetics and evolution. Our research revealed extreme mitochondrial gene rearrangements dominated by gene loss events, potentially leading to the streamlining of *Colpoda* mitogenomes. Surprisingly, while interspecific rearrangements abound, our population-level mitogenomic study revealed a conserved gene order within species, offering a potential new identification criterion. Phylogenomic analysis traced their lineage over 326 million years, revealing two distinct groups. Substantial genomic divergence might be associated with the lack of extended collinear blocks and relaxed purifying selection. This study systematically reveals *Colpoda* ciliate mitogenome structures and evolution, providing insights into the survival and evolution of these vital soil microorganisms.

## INTRODUCTION

As a semi-autonomous organelle, the mitochondrion possesses its own multicopy genome, i.e., mitogenome, which contains important genes related to the electron transfer chain, ribosomal protein synthesis, and other functions (1, 2). Due to their high genetic diversity (3, 4) and rapid evolutionary rate (5), mitogenomes have high precision in identifying highly similar species (6, 7). Multiple copies of mitogenomes in a single cell also make it relatively easy for them to be amplified for sequencing (1, 8–11). Many studies suggest that in some taxa, high spontaneous mutation rates of mitogenomes are one main driver of their high diversity (12, 13). Gene rearrangements of mitogenomes also provide essential clues regarding evolution and ancient relationships (14–16). Although mitochondria have traditionally been thought to be maternally inherited (17), reports from organisms such as *Caenorhabditis elegans* (18) and *Daphnia* (19) have confirmed the presence of considerable heteroplasmy within individual mitogenomes, which has spurred investigations into genome recombination and biparental inheritance in mitochondria (20). Up to now, mitogenomes have been widely studied in biogeography (21), ecology (22), epidemiology (23), and other fields, playing an increasing role in the study of interspecific evolutionary relationships (24–27), population structure, and dynamics (20).

Compared with mitochondrial studies on human diseases and the breeding of animals and plants (28–32), attention to mitochondria shows a much slower growth in ciliates. Since the first reports on mitochondrial DNA of *Tetrahymena thermophila*, *Paramecium primaurelia,* and *Paramecium tetraurelia* in the 1980s (33, 34), mitogenomes of approximately 30 species from the Oligohymenophorea (35), Spirotrichea (36), Heterotrichea (37), and Armophorea (38) classes have been documented. However, this constitutes only a small fraction when considering the tens of thousands of ciliate species, and population-level investigation remains nearly unstudied (39). Unlike the usual circular structure, mitogenomes of ciliates are mostly linear molecules with high AT content, generally possessing telomeric sequences at both ends (1, 34), with gene rearrangements known to occur within the phylum (23). Numerous mitogenes (mitochondrial genes) and the existence of long non-coding sequences including telomeres and central repeats make these mitogenomes of ciliates quite long, generally tens of kilobases. The existence of *ymf* genes, a kind of ciliate-specific mitogenes whose structure and function remain to be explored, also suggests that mitogenomes of ciliates may show different evolutionary characteristics and paths from those of other organisms (4, 40).

Unicellular eukaryotes represent the vast majority of eukaryotic groups (41), and globally distributed ciliated protozoa (42) are considered to be crown eukaryotes that can date back to about 1,980–2,200 million years ago (Mya) in the Paleoproterozoic. Colpodea is a substantial class of ciliates that is about 900 million years old (41, 43, 44). *Colpoda*, a group of ciliates widespread all over the world especially in soil, is a classical genus of Colpodea with kidney-like morphology (45) and unique life history (46, 47). Species in this class show great ecological significance in promoting plant growth and are heavily involved in the biogeochemical cycle (48, 49). As an ancient genus, *Colpoda* has a long evolutionary history. However, the phylogenetic relationships within the genus remain controversial (24, 45, 50). The exploration of *Colpoda* mitogenomes will provide a new perspective on the origin and evolutionary mechanisms of the genus. However, except for several phylogenetic molecular markers such as *cox1*, β-tubulin (24), and partial mito-SSU rDNA (50), mitogenome-level research in the class Colpodea has rarely been reported, which is essential to explore their evolutionary history.

**Fig 1:**
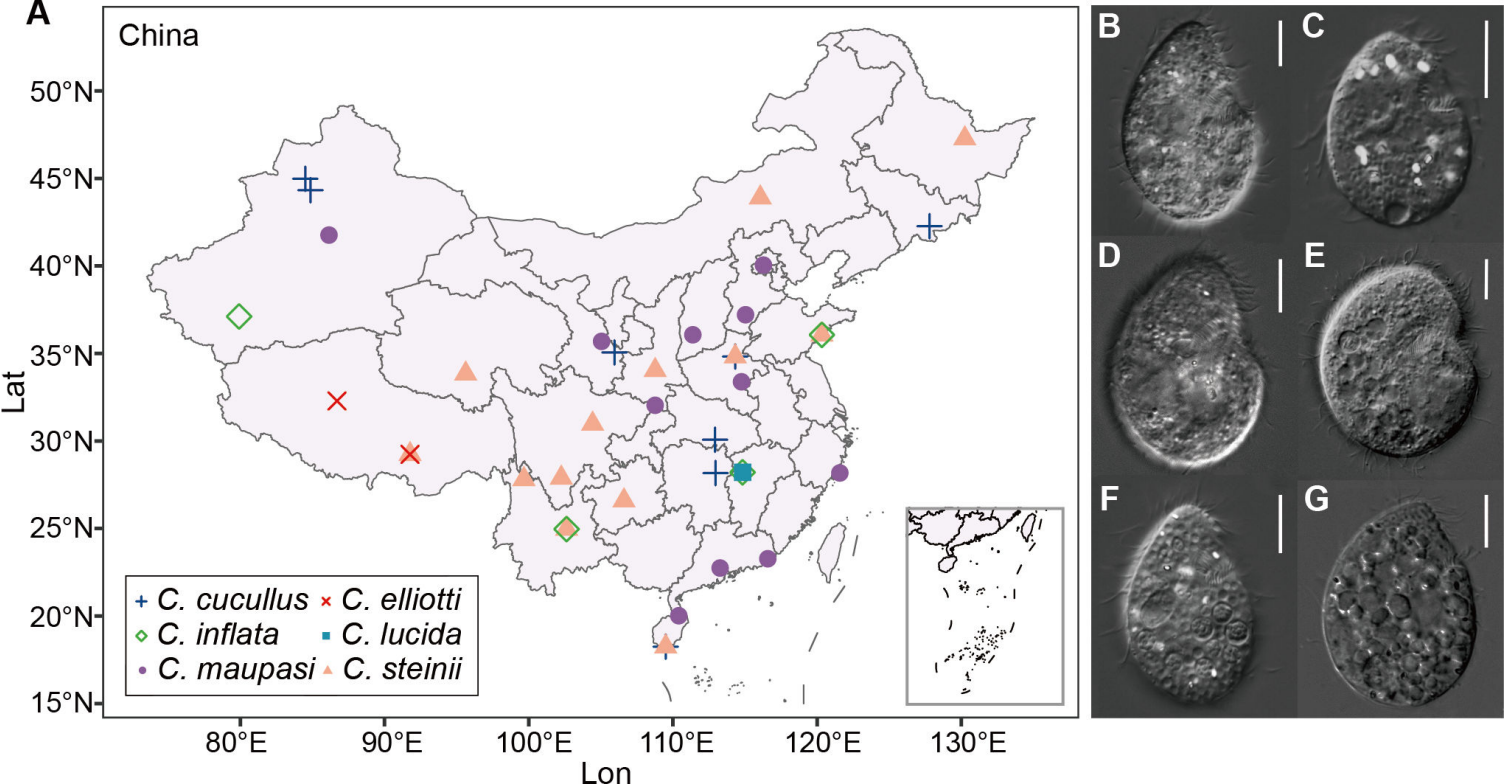
Sampling sites and photomicrographs of the six *Colpoda* species. (**A**) Geographical distribution of collected *Colpoda* strains in China. (**B–G**) Ventral side of *Colpoda cucullus* (MLY2A28), *C. elliotti* (LHA5931), *C. inflata* (RL4B), *C. lucida* (DZG2A41), *C. maupasi* (MW2A28), and *C. steinii* (RZ4A), respectively. The size of the scale bars is 10 µm.

In this study, we isolated 36 natural strains of six *Colpoda* species from topsoil samples collected from China (35 strains; Fig. 1A) and one from Italy. After microscopy, mass culture, omics sequencing of these isolates, and bioinformatics analyses, we *de novo* assembled mitogenomes of six species with 28–33 protein-coding genes (PCGs). By comparing mitogenomic architecture both within and between species of *Colpoda*, we revealed the phylogenomic relationships and divergence time of the *Colpoda* species, identified gene rearrangement events, estimated selection pressure on the mitogenes, analyzed polymorphisms and heteroplasmies in natural strains, and detected linkage disequilibrium signals. Our study deepens the understanding of the mitogenome macroevolution of eukaryotes and provides insights into the speciation of soil microorganisms.

## RESULTS

### *Colpoda* isolation, mitogenome assembly and annotation

Ciliates were isolated from topsoil samples collected from China and Italy. Thirty-six strains of six *Colpoda* species were obtained—*C*. *cucullus* (11 strains), *C. maupasi* (10), *C. elliotti* (1), *C. lucida* (1), *C. inflata* (1), and *C. steinii* (12). The geographical distribution of these species and their morphology are shown in Fig. 1. Additional details are in Table S1.

**Fig 2:**
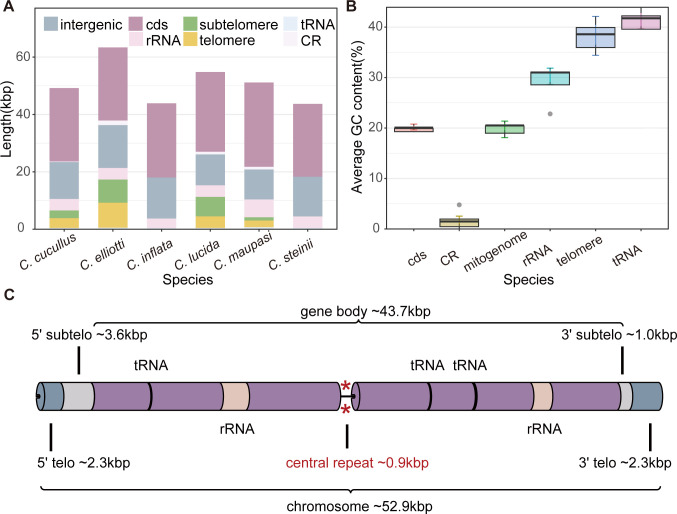
Length, GC content, and blueprint of *Colpoda* mitogenomes. (**A**) Size distribution of different mitogenomic regions in six *Colpoda* mitogenomes. (**B**) Boxplots show the mean GC content of different regions; solid gray circles represent outliers. (**C**) Blueprint of *Colpoda* mitogenomes, visualized by EVenn (52); protein-coding genes are in purple, and their details are shown in Fig. 6.

After long-read sequencing and *de novo* assembly, linear mitogenomes with lengths of 43,691–63,340 bp (mean length of 51,006 bp) and guanine-cytosine (GC) content of 18.11%–21.37% were obtained for each reference strain of the six species (Table 1). The length and GC content of mitogenomic regions with different functional contexts are shown in Fig. 2A and B, respectively. No introns were found. The mitogenomes of *C. cucullus*, *C. maupasi*, *C. elliotti,* and *C. lucida* contain telomeric sequences at both ends. The length of the telomeric repeat units ranges from 32 to 142 bp, with the repeat number ranging from 17 to 273. Contrary to the high telomeric identity observed in mitogenomes of other ciliated genera, no homology was observed between these repeat sequences, suggesting that these telomere sequences have undergone diversifying evolutionary processes or have been independently acquired by different species. For these four species, repeat sequences with high adenine-thymine (AT) content (94.65%–100%) were also found in the mitogenomes and likely constitute the central repetition (CR) regions associated with DNA replication initiation and gene transcription regulation (51). Accordingly, our analysis showed that near CR, the direction of gene transcription usually changes from the forward strand to the reverse one, implying the potential significance of this region in certain yet-to-be-explored transcription regulation mechanisms. Details of telomeres and CRs are given in Table S2. Above all, the structure of the four complete *Colpoda* mitogenomes were elucidated, although we did not acquire telomeric and central repeats in *C. inflata* and *C. steinii*, possibly due to different sequencing strategies or insufficient sequencing depth.

**TABLE 1:** Mitogenomic features of six *Colpoda* species

Species	Strain name	Mitogenome length (bp)	GC content	PCG numbers	tRNA numbers	Sequencing depth
*C. cucullus*	MLY2A28	49,189	21.04%	30	6	137
*C. elliotti*	LHA5931	63,340	20.22%	28	7	220
*C. inflata*	RL4B	43,881	19.32%	30	6	128
*C. lucida*	DZG2A41	54,804	21.37%	30	5	78
*C. maupasi*	MW2A28	51,136	20.80%	33	9	94
*C. steinii*	RZ4A	43,691	18.11%	30	7	80

**Fig 3:**
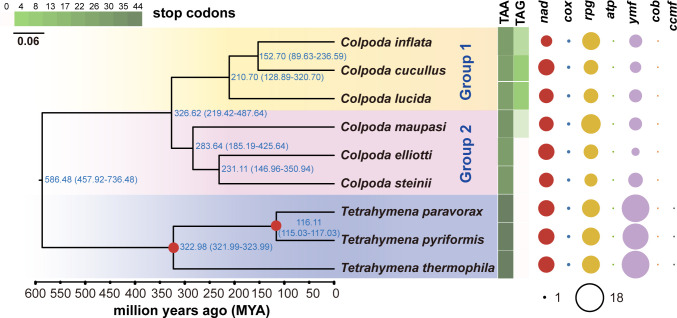
Maximum likelihood (ML) phylogenomic tree based on 22 core genes of the *Colpoda* mitogenomes. The yellow and pink background marks the *Colpoda* species; the blue background marks the outgroup *Tetrahymena*. Numbers beside each node show the estimated divergence time and its confidence intervals in parentheses. The red dots on the nodes indicate the time calibration points based on the divergence time estimation by Xiong et al. (53). The heat map in green gradients represents stop codon usage in all PCGs of each mitogenome, and the bubble map on the right panel displays the types and numbers of mitogenes.

**Fig 4:**
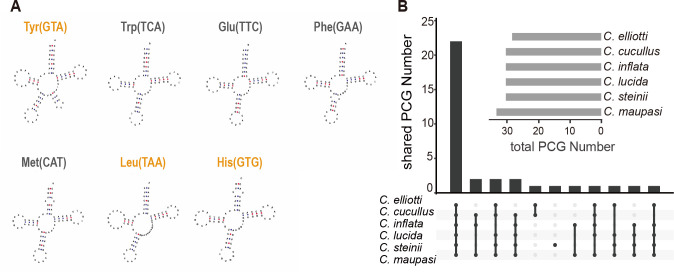
tRNAs and protein-coding gene numbers of *Colpoda* mitogenomes. (**A**) Secondary structures and loss events of *Colpoda* mito-tRNA. Yellow texts indicate the tRNAs that have been lost in the mitogenomes of group 1 (*C. cucullus*, *C. inflata*, and *C. lucida*). (**B**) The horizontal bars in the inset show the total gene number of the mitogenomes; vertical bars show the number of shared PCGs by different *Colpoda* species.

As shown in Fig. 2A, the total length of protein-coding sequences in the six mitogenomes varies from 25,434 to 29,379 bp, accounting for 46.59%–74.39% of the total length (excluding telomeres), which is a relatively low fraction compared to the published ciliate mitogenomes. 28–33 PCGs have been annotated and are assigned to six gene families: complex I: NADH dehydrogenase gene (*nad*), complex III: ubiquinol cytochrome c reductase (*cob*), complex IV: cytochrome C oxidase (*cox*), complex V: ATP synthetase (*atp*), ribosome protein genes (*rpg*), and *ymf*, ciliates-specific genes with high diversity and evolutionary rate (40). *nad1* is split into two parts (*nad1_a*, *nad1_b*) in all *Colpoda* species, as in other ciliates. However, a highly conserved mitogene related to the cytochrome c maturation in ciliates, *ccmf* (51), is not found in *Colpoda* mitogenomes. There are two stop codons in the mitochondrial genomes of *Colpoda*: TAA and TAG, with TAA being the main stop codon of *Colpoda* and the only one in the *Tetrahymena* mitogenome, while stop codon TAG is used less but exists widely in four *Colpoda* species (Fig. 3). As in other ciliates, two mito-rRNA genes were annotated in all six *Colpoda* species, but the number of tRNAs varies from 6 to 9 among different species. All of the latter possess the typical four-armed cloverleaf secondary structure, as shown in Fig. 4A.

### Phylogenomic relationships and evolutionary history of the *Colpoda* genus

Homologous gene analysis by Orthofinder v2.5.4 revealed 35 orthogroups (Fig. 4B), of which 22 were core genes that existed in all six *Colpoda* mitogenomes: *nad1*, *nad3, nad4*, *nad4L*, *nad6*, *nad7, nad10*, *cox1*, *cox2*, *cob*, *atp9*, *rpl2*, *rpl16*, *rps12*, *rps13, rps14*, *rps19*, *ymf57*, *ymf65*, *ymf66*, *ymf67,* and *ymf68*. Additionally, the homology analysis showed that certain *ymf* genes were homologous to some ribosomal protein genes, i.e., *ymf60* was homologous to *rpl6*, *ymf59* to *rps10*, and *ymf76* to *rps4*.

As shown in Fig. 3, the phylogenomic tree based on concatenated core gene sequences showed that *Colpoda* species form two groups, with *C. cucullus*, *C. inflata,* and *C. lucida* as the first group and another three species as the second. Although our results were slightly different from those of previous studies, such as the closer relationship of *C. inflata* and *C. cucullus* in this study vs *C. lucida* and *C. cucullus* in Foissner et al. (45), this may be caused by different input genes and length and tree construction methods. High bootstrap values, which are shown in Fig. S1, indicate the reliability of core mito-PCGs in reconstructing the evolutionary relationship of *Colpoda* species.

The molecular clock is a dependable technique for gauging the rate of organism evolution, which relies on the premise that genomes accumulate neutral mutations at a consistent rate throughout time. The extrapolation of the divergence time based on phylogenomics calibrated by the molecular clock indicates that *Colpoda* diverged from the lineage leading to *Tetrahymena* about 586 Mya during the Proterozoic period (Fig. 3). At least 326 Mya, i.e., the Carboniferous period, when nearly 50% of the animals on land experienced extinction (54), *Colpoda* diverged into two lineages. The second lineage began to diverge within the next 43 million years or so, and it fully diverged into three species: *C. maupasi, C. steinii,* and *C. elliotti* within the next 52 million years or so. The ancestors of the first lineage took 116 million years to diverge into *C. lucida*, and finally, *C. inflata* and *C. cucullus* originated about 152 Mya. We verified the reliability of the inferences by two independent runs for the divergence time (*R*^2^ = 1.00, Fig. S2).

### High mitogenomic divergence within the *Colpoda* genus

**Fig 5:**
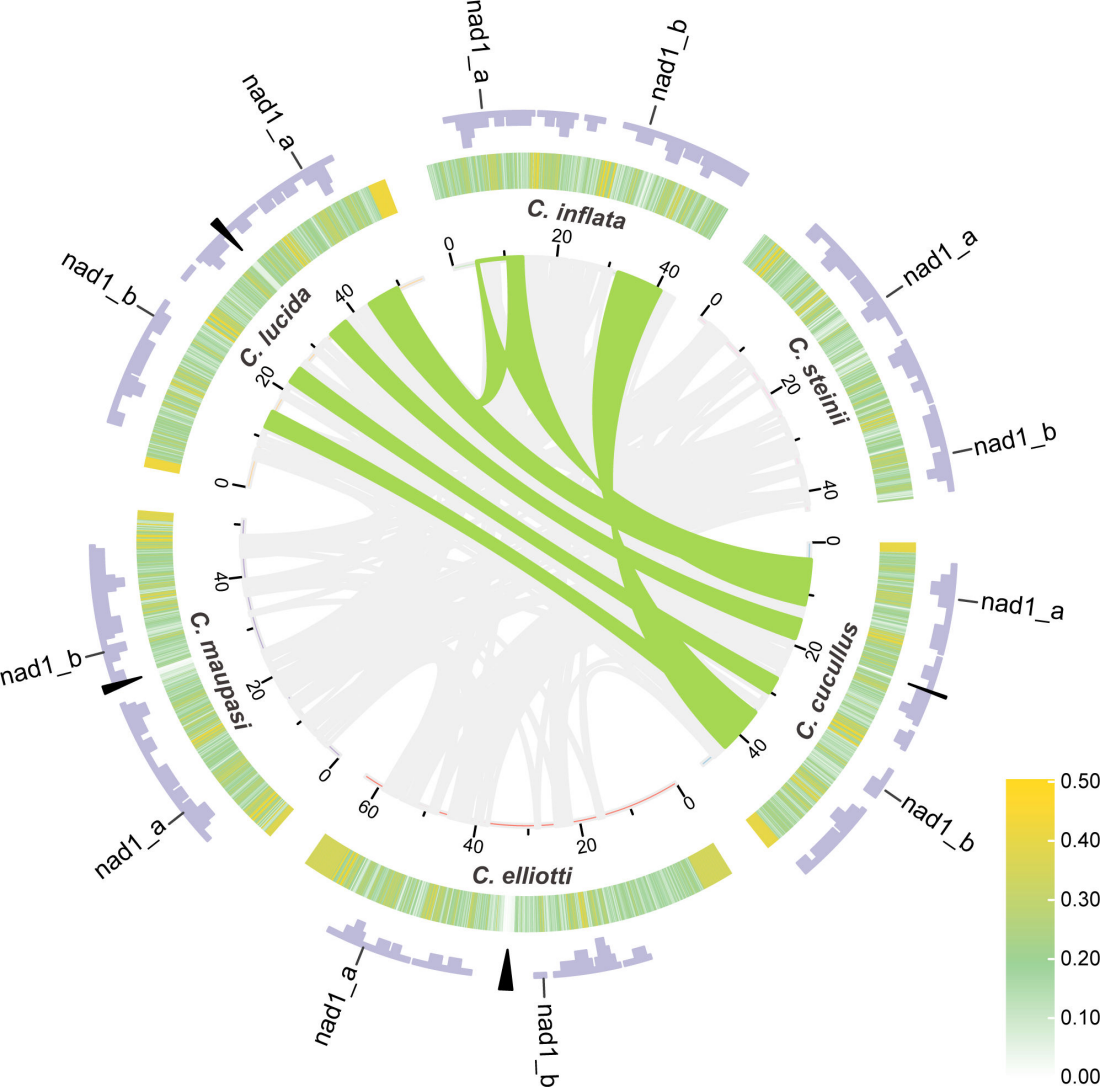
Collinearity of *Colpoda* mitogenomes. The lines connecting different species in the central circle represent collinearity, gray lines show synteny blocks less than 2.5 kbp, and green lines show synteny blocks greater than 2.5 kbp. The tick marks represent the coordinates of the mitogenomes in kilobase pair. The heat map in the middle track shows the GC content (window size = 0.1 kbp, step size = 0.05 kbp). The outmost purple bars show gene density (window size = 1 kbp, step size = 0.5 kbp), the black triangles show the locations of the CR regions, and nad1_a and nad1_b indicate two parts of the split gene *nad1*.

Using collinearity analysis, we identified many syntenic blocks among mitogenomes within the genus. As shown in Fig. 5, these collinear blocks avoid the regions where the telomeres and CRs are located; there are also some gaps in the remaining regions, primarily consisting of intergenic sequences and a few genes with low sequence identity. The total length of collinearity blocks between species pairs varies largely from 8.50 kbp (*C. elliotti* and *C. inflata*) to 33.50 kbp (*C. cucullus* and *C. inflata*). Overall, the interspecies collinearity block in group 1 is the largest, with the mean length of 30.25 kbp, followed by 17.79 kbp of group 2. The synteny degree between the two groups is the lowest, which is just 14.0 kbp in length. For single collinear blocks, most of them are less than 2.50 kbp in length, suggesting that long continuous collinear sequences may have been interrupted by frequent evolutionary events such as gene rearrangements. All seven blocks longer than 2.50 kbp are from species within the first group, possibly due to closer relatedness between species in this clade.

### Unusual gene rearrangement patterns in *Colpoda* mitogenomes

**Fig 6:**
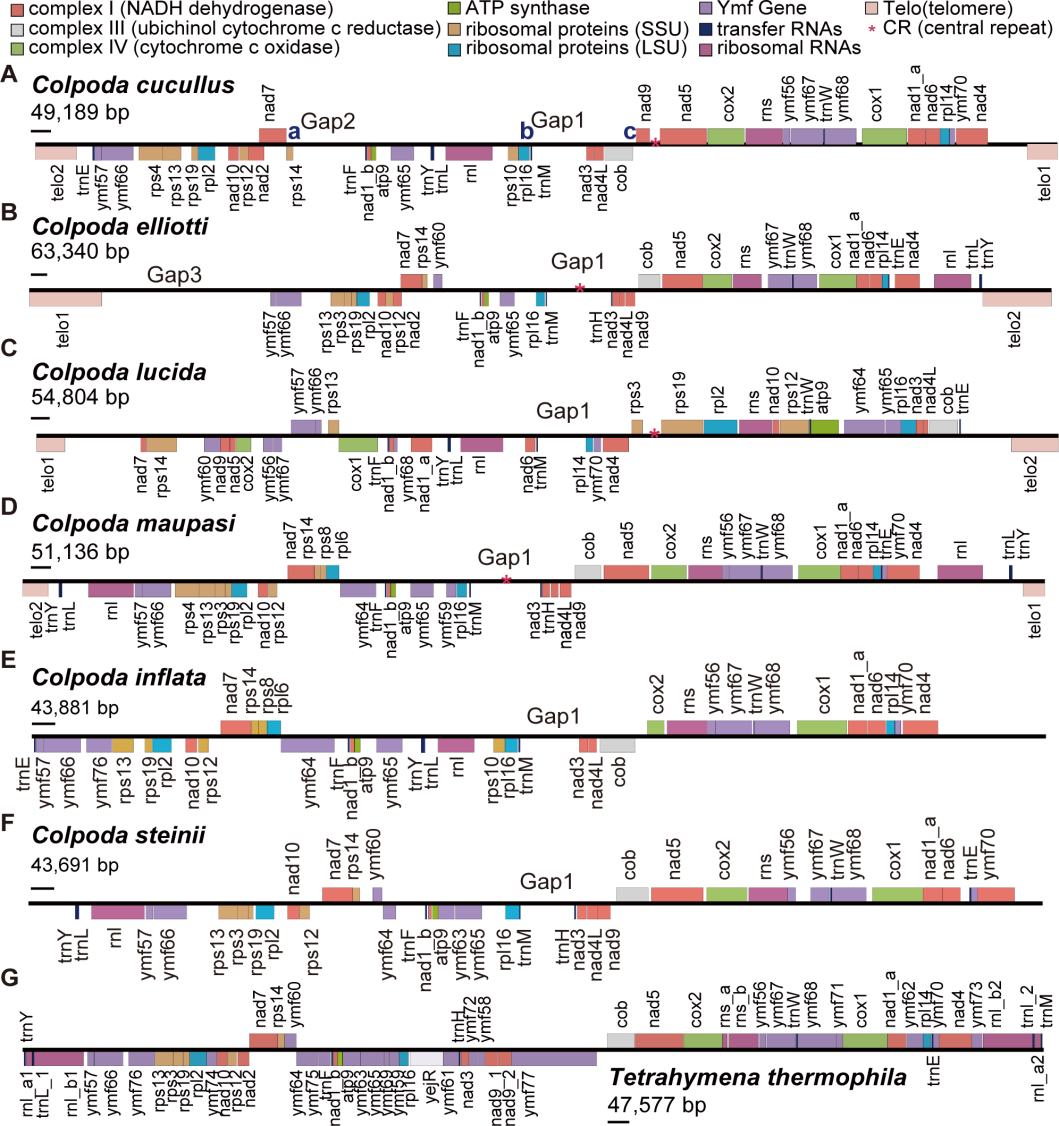
Gene orders of *Colpoda* spp. vs *Tetrahymena thermophila* mitogenomes. (**A–F**) The gene contents of six *Colpoda* mitogenomes. Blue letters indicate three rearrangement hotspots in *C. cucullus*. (**G**) Organization of the *Tetrahymena thermophila* mitogenome (55). The short horizontal bars beneath the genome sizes are scale bars of 1 kbp.

Generally, the mitogenome structure is unlikely to be totally identical in class-level phylogenetic comparison, although gene orders of mammals, insects, and fish are relatively conservative (14). However, ciliate mitogenomes show a unique organization compared with other organisms. Usually, they show high agreement in gene order at the genus level but lack a uniform pattern within the whole phylum. In striking contrast, *Colpoda* mitogenomes show a nearly scrambled gene order between congeners, leading to a no consensus mitochondrial gene order for the genus (Fig. 6). Given the resemblance of mitochondrial gene order between *C. maupasi* and most non-*Colpoda* species in the class Oligoheymenophorea, we established the gene order of *C. maupasi* as the reference standard and conducted comparisons with other *Colpoda* species to quantify the extent of rearrangements among congeners.

**Fig 7:**
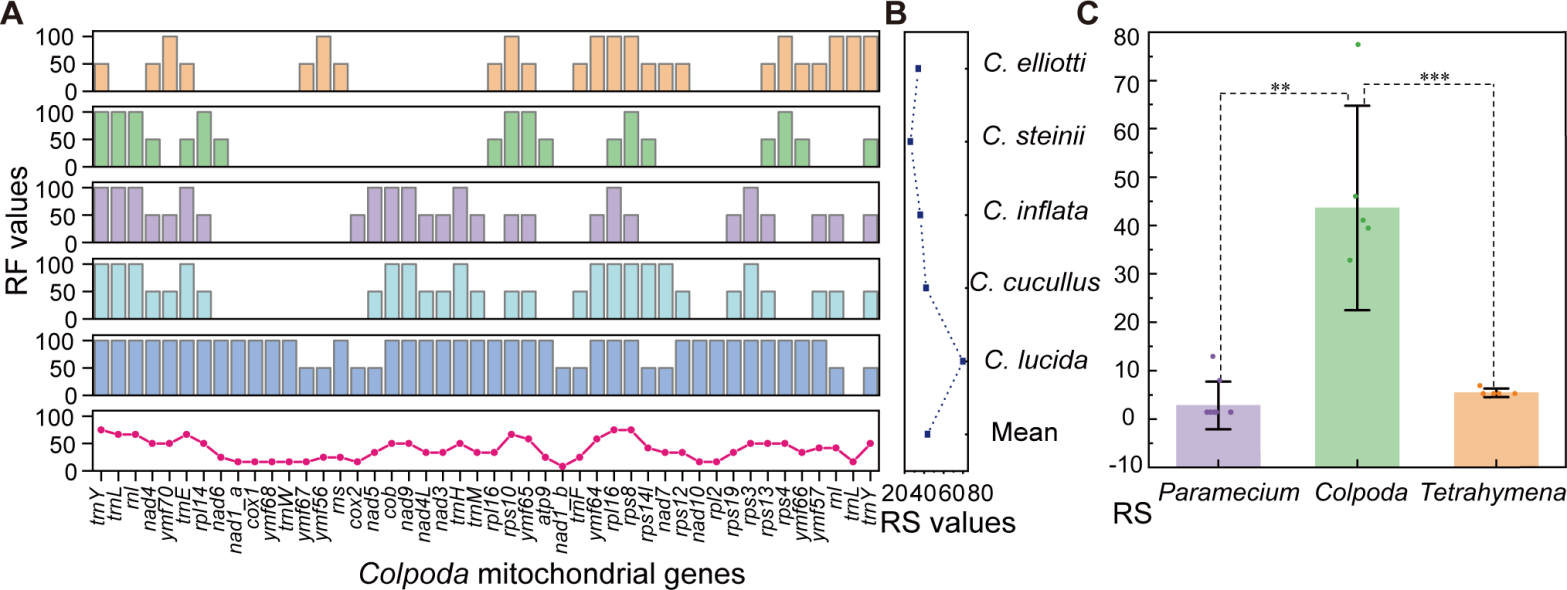
Rearrangement estimation of *Colpoda* mitogenomes. (**A**) Upper five histograms show distribution of RF of mitogenes in different species. The line plot at the bottom shows the mean RF value per gene across the five *Colpoda* species. The gene order of *C. maupasi* was used as the baseline reference. (**B**) RS of six *Colpoda* mitogenomes. (**C**) *t*-tests of RS values for *Colpoda*, *Paramecium*, and *Tetrahymena*. ** indicates a *P*-value <0.01; *** means a *P*-value <0.001.

We used qMGR (56) to quantify the rearrangement events that cause mitochondrial gene order variation within *Colpoda*, which assigns equal weight to different types of rearrangement events (i.e., gene deletion, inversion, and translocation). The results are shown in Fig. 7A. Among all genes, the mean rearrangement frequency (RF) of tRNA is 47.91, and that of PCGs is 37.14, indicating that tRNA genes are more often involved in gene rearrangements. *trnY-trnL-rnl* is the most frequently rearranged hotspot, with a mean RF of 69.40. The most conserved gene block is *nad1_a-cox1-ymf68-trnW-ymf67*. Overall, regardless of the greater overall sequence divergence, the three species in group 2 that diverged earlier have experienced fewer rearrangement events [mean rearrangement score (RS) = 20]; that is, they may retain more ancestral gene order patterns. Mitogenomes of *C. inflata* and *C. cucullus* of group 1 rearranged more actively, with RS of 36 and 42 (Fig. 7B), respectively. As an exception, in the *C. lucida* mitogenome, all genes except *trnL* experienced at least one rearrangement event, elevating the total RS value to 80, which was the highest among all six species.

According to the above analyses, the gene orders of mitogenomes in different *Colpoda* species are extremely scrambled by rearrangement events. Gene losses are the most dominant rearrangement events in the mitogenomes of *Colpoda,* and the degree of rearrangements is not necessarily associated with species relatedness (Fig. 7A). In contrast, after filtering out low-quality *de novo* mitogenome assemblies of strains belonging to three species (*C. maupasi, C. cucullus,* and *C. steinii*) short-read-sequenced, we observed that the mitogene orders within nine *C. steinii* strains remained highly conserved, as shown in Fig. S3.

We also investigated gene rearrangement events in other ciliate mitogenomes, as shown in Fig. 7C and Table 2. The results of *t*-tests revealed an extreme increase in the RS of *Colpoda* mitogenomes (mean = 43.60, *P* < 0.05), compared to the conserved pattern observed in *Tetrahymena* (5.40) and *Paramecium* (2.80). After taking into account of estimates from most previous reports, we also found that *Colpoda* mitogenomes are one of the highest in rearrangement scores among mitogenomes of all studied eukaryotes (Table 2), although the selection of benchmark genome might somewhat affect the RS score. Future efforts on normalization and consideration of lineage depths may also be needed for more accurate estimation.

**TABLE 2:** Rearrangement scores of mitogenomes in different organisms

Genus or lineage	Species number	RS (mean)	RS (SE)	Reference
Fishes	2,558	0.13	~0.91	(56)
*Tetrahymena^a^*	6	5.40	2.14	(35, 40, 51, 55)
Mammals	1,055	0.24	~1.58	(56)
Amphibians	242	1.52	~2.83	(56)
*Paramecium* ^*a*^	11	2.80	4.52	(20, 51)
Reptiles	305	3.22	~4.41	(56)
Birds	614	5.85	~0.60	(56)
Fungi^*a*^	38	32.95	2.13	(57)
*Colpoda^a^*	6	43.60	23.70	This study
*Acari^a^*	5	69.40	3.88	(58)

^*a*^ Indicates the RS values calculated in this study.

### Widespread relaxed purifying selection

**Fig 8:**
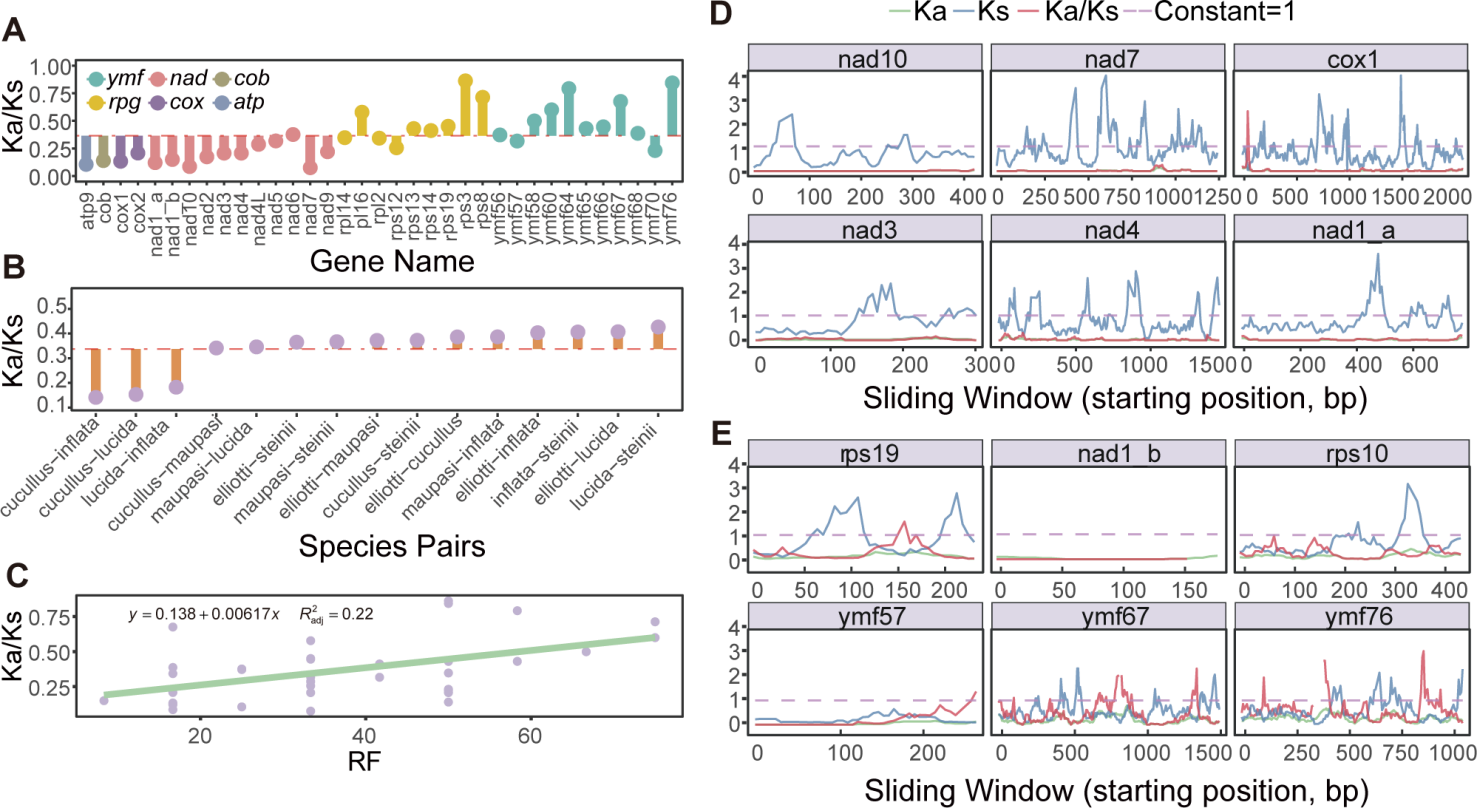
Selective pressure on *Colpoda* mitogenes. (**A**) Lollipop chart shows mean Ka/Ks values of each homologous gene. The red dotted line represents the mean value. (**B**) Mean Ka/Ks values of each species pair. (**C**) Non-significant correlation between RF and selection pressure (Ka/Ks). The Ka/Ks values increase from left to right. (**D–E**) Changes in Ka, Ks, and Ka/Ks values of six gene pairs with minimum and maximum Ka/Ks values of *C. cucullus* and *C. inflata*, corresponding to green, blue, and red, respectively. *X* coordinates represent the starting site of each sliding window; the purple dashed lines represent constant 1.

To explore the natural selection on mitogenomes of the *Colpoda* genus, we conducted an analysis of selection pressure on 35 orthogroups in six *Colpoda* mitogenomes by ParaAT v2.0 and KaKs_Calculator v2.0. As shown in Fig. 8A, the Ka/Ks values of all genes are less than 1, with a mean value of 0.37, indicative of universal purifying selection. Among all PCGs, *nad7* was the most conserved, with the lowest Ka/Ks value (0.08), while *rps3* had the highest Ka/Ks value (0.86). We also found that to a large extent, the genes directly involved in the electron transport chain of aerobic respiration are highly preserved by purifying selection (Fig. 8A). For example, those in complex V (*atp*) were the most conserved with a Ka/Ks value of 0.11, followed by complex III (*cob*), complex IV (*cox*), and complex I (*nad*) genes with values of 0.14, 0.17, and 0.20, respectively. As two groups of genes that account for more than half of the *Colpoda* mitogenomes, *rpg* genes, which are involved in ribosomal protein synthesis, and the *ymf* genes, which have the highest variability, their mean Ka/Ks values are as high as 0.49 and 0.51, which are about three times that of Ka/Ks values of the aforementioned four genes in the electron transport chain, thus experiencing less selective constraint and contributing greatly to the high overall mitogenome divergence. This is also consistent with the conclusion proposed before that *ymf* genes of *Tetrahymena* are mutation hotspots (40). Figure 8B shows the Ka/Ks values among 15 species pairs. The mean Ka/Ks is 0.33. The lowest value is 0.13 of *C. cucullus* and *C. inflata*, followed by *C. cucullus–C. lucida* and *C. lucida–C. inflata* pairs, which confirmed the lower degree of differentiation among group 1 species. The highest Ka/Ks value of 0.42 is found in the *C. lucida–C. steinii* pair. A sliding-window method was also used to show the distribution of Ka/Ks values of each gene pair in more details (Fig. 8D and E). There was not any significant correlation between selection strength and the frequency of rearrangement events (Fig. 8C; *R*^2^ = 0.22, *P* = 0.0028).

### Polymorphisms and heteroplasmies inferred from population genomics of *Colpoda*

**Fig 9:**
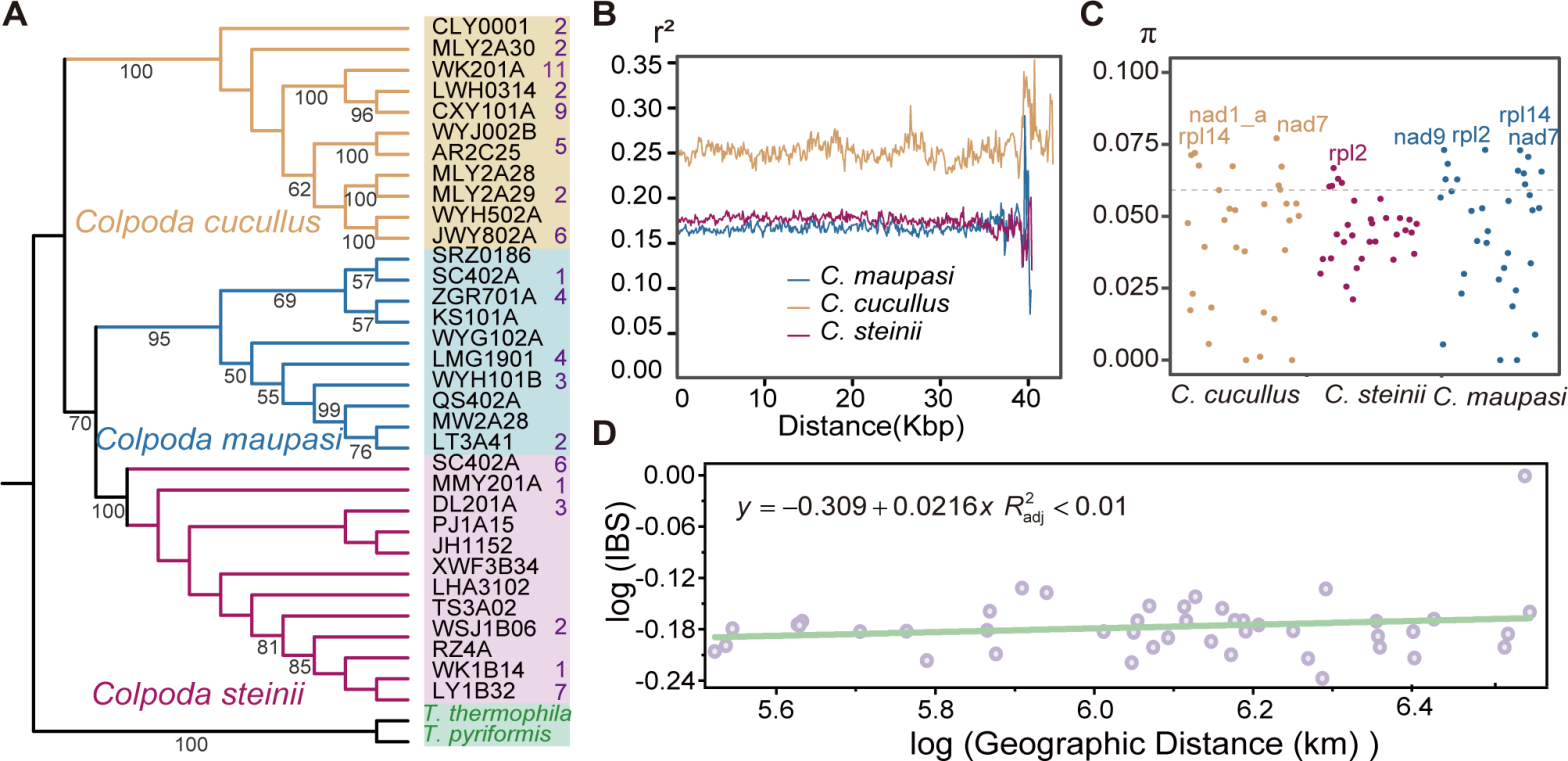
Population genetic analyses on natural strains of *C. cucullus*, *C. maupasi,* and *C. steinii*. (**A**) An ML phylogenetic tree was constructed based on 1,588 nucleotide sites of the mitochondrial small subunit rRNA gene of 33 *Colpoda* strains. Species are represented by strain names in black; the outgroups are *T. pyriformis* (AF160864.1) and *T. thermophila* (AF396436.1). Only bootstrap values >50 are shown. The rightmost numbers in purple are numbers of heteroplasmic sites. (**B**) Relationships between the linkage disequilibrium index (measured by *r*^2^) and physical distance between sites. *X*-axis numbers are mitogenomic coordinates of the genes. (**C**) Nucleotide diversity of the PCGs of three *Colpoda* species. The dotted line represents the third quartile of π value 0.059. (**D**) Scatter plot of isolation by state (IBS) versus geographic distances (in kilometer) among 10 *C. maupasi* strains. Mantel test for matrix correlation between log (IBS) and log (geographic distance): *R*^2^ < 0.01.

The median of the sequencing depth for the 33 natural strains of *C. cucullus*, *C. maupasi,* and *C. steinii* is 68×, and the median of coverage breadth is 79.20% (Table S1). The mito-SSU rRNA single-gene tree shows the expected clustering of strains of the same species, demonstrating high species delimitation, and the phylogenetic distance of *C. maupasi* and *C. steinii* is closer than that with *C. cucullus*, which is consistent with the multi-gene phylogenetic inference and highlights the potential of this gene for species identification (Fig. 3 and 9A). After SNP calling and filtering, we identified in total 3,262, 3,752, and 4,408 SNP sites within the mitogenomes of *C. cucullus, C. maupasi*, and *C. steinii*, respectively. This corresponds to densities of 66, 73, and 101 SNP sites per kilobase pair (Fig. S4). Potential heteroplasmy was then examined within each strain of *Colpoda*. Among the strains of *C. cucullus* (11), *C. maupasi* (10)*,* and *C. steinii* (12), seven, five, and six strains, respectively, were identified with potential heteroplasmic loci. The cumulative number of heteroplasmic sites for each species was 37, 14, and 20, respectively (Fig. 9). However, after the LD decay test, no significant correlation was observed between the value of *r*^2^ and the distance separating the sites, although our estimates of *r*^2^ values may be on the high side due to the influence of, e.g., mutation hotspots. This lack of correlation implies that recombination is rare within *Colpoda* mitogenomes (Fig. 9B).

Despite the presence of heteroplasmic sites, a single consensus sequence was obtained for each strain based on the major alleles. Using these consensus sequences, we calculated the nucleotide diversity of the mitogenomes between individuals of each species, resulting in mean π values of 0.04, 0.05, and 0.05, respectively (Fig. 9C; Fig. S4, track d). Notably, certain genes deviating from the norm are labeled in Fig. 9C. In order to explore the role of geographical distance in the formation of genetic diversity, a correlation analysis between IBS and individual geographic distance was conducted, and the results are shown in Fig. 9D. Strains within the genus do not show an obvious geographic–genetic pattern on the whole, and geographical factors do not appear to cause universal restriction of gene flow in *Colpoda* mitogenomes. Figure 9D shows the association analysis between the 10 *C*. *maupasi* strains, and the same geographic–genetic independence could also be found in *C. cucullus* and *C. steinii* (Fig. S5).

## DISCUSSION

In this study, we conducted a systematic analysis of the mitogenomes of six *Colpoda* species, including 36 natural strains isolated from over a large geographical range, taking advantage of intensive sample collection, microscopy, genome/transcriptome sequencing, and omics and evolutionary analyses. After complete mitogenome assembly and annotation, structures and evolution of mitogenes were elucidated. Phylogenomic analysis revealed the ancient origin of the genus, and the low degree of collinearity indicated substantial divergence between *Colpoda* mitogenomes. During evolution, mitogenomes of different *Colpoda* species have experienced extremely active rearrangement events, while the gene order between strains of the same species still exhibited high stability. This might suggest a high fidelity in mitochondrial inheritance within species and the presence of robust genetic boundaries between different species. Selection analysis demonstrated that although the coding regions of the *Colpoda* mitogenome experienced purifying selection, the selection pressure was weaker in over half of the genes (*rpg, ymf*) than those related to the electron transport chain of aerobic respiration (*nad, atp, cox,* and *cob*). Population-level analyses detected variation between (polymorphisms) and within (heteroplasmies) strains, as well as revealed no recombination and non-association between geographic and genetic distances. Nonetheless, it is crucial to acknowledge that the accuracy of our sequenced clones in reflecting the actual levels of intraspecific diversity may be influenced by the limited number of strains we studied and the geographic restrictions in our sampling.

The long-read sequencing technology enabled us to obtain near-complete mitochondrial genomes at a telomere-to-telomere scale for four of the six *Colpoda* species in this study. After investigating more than 20 published ciliate mitogenomes, we found that most of the repeat units of telomeres are shorter than 55 bp in length. Among them, those in *Tetrahymena thermophila* and *Miamiensis avidus* (Oligoheymenophorea) are 53 bp (33) and 30 bp (59), respectively. In the ciliate class Spirotrichea, such as *Strombidium* sp., *Sphaerium* cf. *sulcatum*, *Laurentiella strenua*, *Oxytricha trifallax,* and *Halteria grandinella,* they are 18, 34, 36, 35, and 31 bp, respectively (4, 36). By contrast, *Colpoda* have significantly longer telomeric repeat units without homology between species and higher GC content. Among them, *C. maupasi* has an extremely long telomere repeat unit with a length of 142 bp, followed by *C. lucida* and *C. cucullus,* with 76 and 65 bp, respectively, and finally *C. elliotti* with 32 bp.

Studies have shown that telomere structures can shield the exposed chromosome ends of telomeric DNA from DNA damage (60). In this study, except for *C. maupasi* (21.78%), the telomere sequences of the other three *Colpoda* species showed higher GC content (mean = 38.82%, SE = 0.03) than those of other ciliates (Table S2). This may correspond to a more stable nucleotide primary structure, which could play a role in protecting genes in the mitogenome. Surprisingly, the total length of *C. elliotti*’s telomeres reached 8.75 kbp, accounting for 13.70% of the total mitogenomic length, which is the longest telomere sequence ever reported in ciliates. Because this strain was collected from Tibet with high altitude and low oxygen content, we speculate that the extremely long telomeres are associated with the extra protection of the mitogenomes from especially harsh environmental conditions, although more empirical evidence on this is needed.

Based on the phylogenomic analysis, we estimated that *Colpoda* originated more than 326 Mya, which is highly similar to that of *Tetrahymena* (about 323 Mya) (53). Collinearity and other analyses further showed that *Colpoda* mitogenomes had higher genome diversity, particularly in the group 2 species, suggesting that the mitogenomes of *Colpoda* may have undergone extraordinarily rapid evolution.

We found that gene loss dominated the rearrangement events in *Colpoda* mitogenomes, with the fragments of these lost genes remaining in the mitogenome, resulting in long non-coding regions between certain genes. For example, as shown in Fig. 6A through D, Gap1 was caused by the loss of *ccmf* gene, and Gap2 in *C. cucullus* was caused by the loss of *ymf64* and *ymf75*. In diploid nuclear genomes, gene deletion events generally occur after whole-genome duplication in an organism, leading to the loss of some functional genes (61). To explain the gene order change in mitogenomes, Boore and Brown (62) proposed the tandem duplication–random loss model, which attaches great importance to the duplication of mitogenes followed by gene loss. They indicated that by pseudogenization and degeneration of the redundant genes, gene orders were scrambled in different patterns. While this model was generally used to explain similar gene orders, more mechanisms are needed to explain the extremely scrambled genes in *Colpoda* mitogenomes. Furthermore, the highly conserved intraspecific and extremely variable interspecific gene orders of the mitogenomes may contribute to reliable *Colpoda* species identification, by adding more evidence besides morphological traits and conservative gene barcoding. To determine the applicability of this concept to a wider range of taxa, further population-level investigations across diverse ciliate species are required.

Gene rearrangement hotspots may also accelerate gene order scrambling. For example, Montoya et al. (63) found that near mito-*SSU rRNA* in the human mitogenome, genes experienced more rearrangements. Similarly, the mean RF of *LSU rRNA-Leu-Tyr* block of *Colpoda* reached as high as 52.80, and this might be caused by a similar mechanism associated with large subunit rRNA (*rnl*). Additionally, Boore and Brown (62) proposed that near the origins of replication, gene rearrangements are extremely active, which is highly consistent with the high RS value of the sub-CR block *nad9-cob* (RS = 50). Gene orders could also be shaped by selection (64), though in *Colpoda* mitogenomes we only found a weak correlation between RS and Ka/Ks values (Fig. 8D).

Besides the extremely scrambled gene order, some unknown genes were also found in the *Colpoda* mitogenomes. For example, near the 5′ end of the *C. elliotti* mitogenome (Fig. 6B, Gap3), a sub-telomeric sequence of 10.45 kbp was identified, but the lost *trnY-trnL-rnl* block here was just about 1.9 kbp in length, which was uncommon in the tightly packed ciliate mitogenomes. Although this sequence does not align with any known gene, about 50 open reading frames greater than 75 nt in length were identified by ORFfinder (65), and RNAseq data also supported that four of them have transcripts (Fig. S6). Among them, the longest is 825 aa (+ strand; hypothetical protein 1), followed by 440 aa (− strand; hypothetical protein 2), 132 aa (+; hypothetical protein 3), and 90 aa (+; hypothetical protein 4), and start at positions of 4,554, 8,400, 10,132, and 11,816 bases from the 5′ end, all with TAA as the stop codon (Fig. S6). Further empirical investigations are required to determine their specific functions.

In addition, some known mitogenes of other ciliates are absent from *Colpoda* mitogenomes, such as the cytochrome C maturation protein gene (*ccmf*). *ccmf* is essential for the aerobic mitochondria of eukaryotes as it is associated with the maturation of cytochrome c, which binds to heme and transfers electrons between respiratory chains (66). Deletion of the *ccmf* gene may be a consequence of its relocation into the nuclei, but a partial remnant of this gene is present in *Colpoda* mitogenomes, which suggests that it may have gradually lost its function under relaxed selection pressure or the function was replaced by nuclear genes. Using *ccmf* and *ymf* genes as examples, it remains unclear whether the frequent gene loss events in *Colpoda* mitogenomes imply the elimination of redundant genes and if the aerobic respiration process of *Colpoda,* which inhabit the soil, is altered compared to that of typical aquatic ciliates.

To conclude, we have identified extremely high gene rearrangement events in the mitogenomes of *Colpoda*, one of the most widely distributed ciliates. Considering their unique habitat (almost exclusively in the soil) and the scrambled gene orders compared to other ciliates, these rearrangements may shed light on their capacity to adapt to the harsh and ever-changing soil environments they inhabit. Moreover, the streamlined mitogenomes, possibly stemming from these extensive rearrangements, suggest natural selection for energy efficiency, achieved by shedding unnecessary genetic baggage, while simultaneously facilitating broader dispersal. However, the intricacies of evolutionary strategies may extend beyond the organelle level, prompting us to embark on an ongoing exploration of *Colpoda*’s macronuclear genomes. This endeavor promises to provide deeper insights into the mysteries surrounding these highly successful and prolific denizens of the soil and possibly even give clues to the evolution of other single-celled eukaryotic organisms.

## MATERIALS AND METHODS

We newly isolated six *Colpoda* species, primarily sourced from the top 15 cm of mostly mainland China’s soils (Table S1), resulting in the acquisition of 36 natural strains. These species were identified through 18S rRNA sequencing, with the cutoff value set at >99% sequence identity. Nucleic acids were extracted using the MasterPure Complete DNA and RNA Purification kit. To obtain comprehensive mitogenomic data, we employed long-read sequencing technologies, such as the PacBio HiFi and Nanopore MinION platforms, on reference strains of the six species. Additionally, we conducted Illumina NovaSeq6000 PE150 deep sequencing for all 36 strains.

To further investigate transcription patterns, single-cell RNA sequencing was performed on *C. elliotti*. Detailed procedures for mitogenome assembly and annotation, collinearity and phylogenomics analyses, gene rearrangements, and population genetics can be found in the Supplementary Materials and Methods section.

## Data Availability

Assembled mitogenomes and annotations are available for access via the National Genomics Data Center of China (https://ngdc.cncb.ac.cn/). The corresponding accession numbers are as follows: C_AA048790.1, C_AA048789.1, C_AA048788.1, C_AA048787.1, C_AA048786.1, C_AA048785.1. Furthermore, the raw sequencing data are under BioProject PRJNA1010524 on NCBI (https://www.ncbi.nlm.nih.gov/), encompassing PacBio HiFi sequencing FASTQ files for reference strains, Illumina sequencing FASTQ files for population strains (see details in Table S1), and FASTQ files for low-input RNA-seq data. The Nanopore sequencing data for C. inflata (RL4B) and C. steinii (RZ4A) can be accessed through NCBI BioProject study numbers SRR23588191 and SRR23588197.

## SUPPLEMENTAL MATERIAL

Supplemental Figures msystems.01161-23-s0001.pdf Figures S1-S6.

Supplemental text msystems.01161-23-s0002.pdf Details of the materials and methods.

Supplemental Tables msystems.01161-23-s0003.pdf Tables S1 and S2.

ASM does not own the copyrights to Supplemental Material that may be linked to, or accessed through, an article. The authors have granted ASM a non-exclusive, world-wide license to publish the Supplemental Material files. Please contact the corresponding author directly for reuse.
