# Supplementary material for: Mitogenomic architecture and evolution of the soil ciliates Colpoda
Source: mSystems. 2024 Jan 23;9(2):e01161-23. doi: 10.1128/msystems.01161-23 (PMC10878089; doi:10.1128/msystems.01161-23)
Supplement: Supplemental Figures — Figures S1-S6. [file msystems.01161-23-s0001.pdf]

Mitogenomic architecture and evolution of the soil ciliates *Colpoda*

Yuanyuan Zhang[1,2], Haichao Li[1], Yaohai Wang[1], Mu Nie[1], Kexin Zhang[1], Jiao Pan[1], Yu Zhang[1,3], Zhiqiang Ye[4], Rebecca A. Zufall[5], Michael Lynch[6], Hongan Long[1,2,*]

1. Key Laboratory of Evolution and Marine Biodiversity (Ministry of Education), Institute of Evolution and Marine Biodiversity, KLMME, Ocean University of China, Qingdao, Shandong Province, China 266003
2. Laboratory for Marine Biology and Biotechnology, Laoshan Laboratory, Qingdao, Shandong Province, China 266237
3. School of Mathematics Science, Ocean University of China, Qingdao, Shandong Province, China 266000
4. School of Life Sciences, Central China Normal University, Wuhan, Hubei Province, China 430079
5. Department of Biology and Biochemistry, University of Houston, Houston, Texas, USA 77204
6. Biodesign Center for Mechanisms of Evolution, Arizona State University, Tempe, Arizona, USA 85287

*Corresponding author: longhongan@ouc.edu.cn

FIG S1
FIG S2
FIG S3
FIG S4
FIG S5
FIG S6

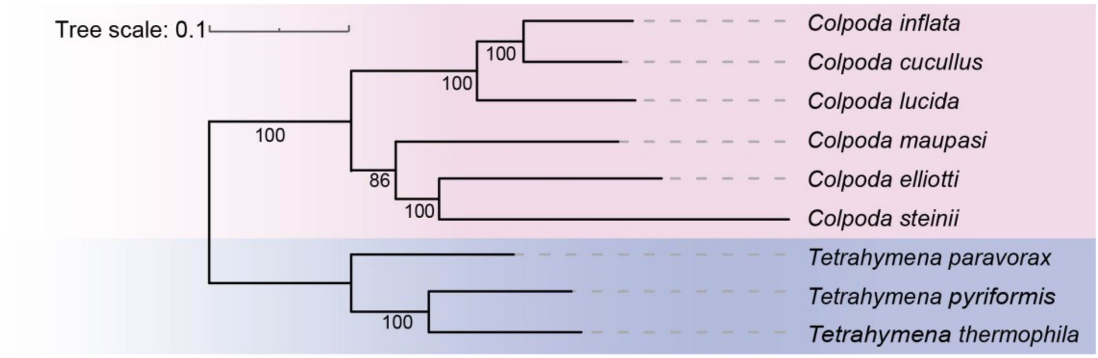

FIG S1 ML phylogenomic tree of *Colpoda* based on 22 mito-genes. Numbers show the bootstrap values.

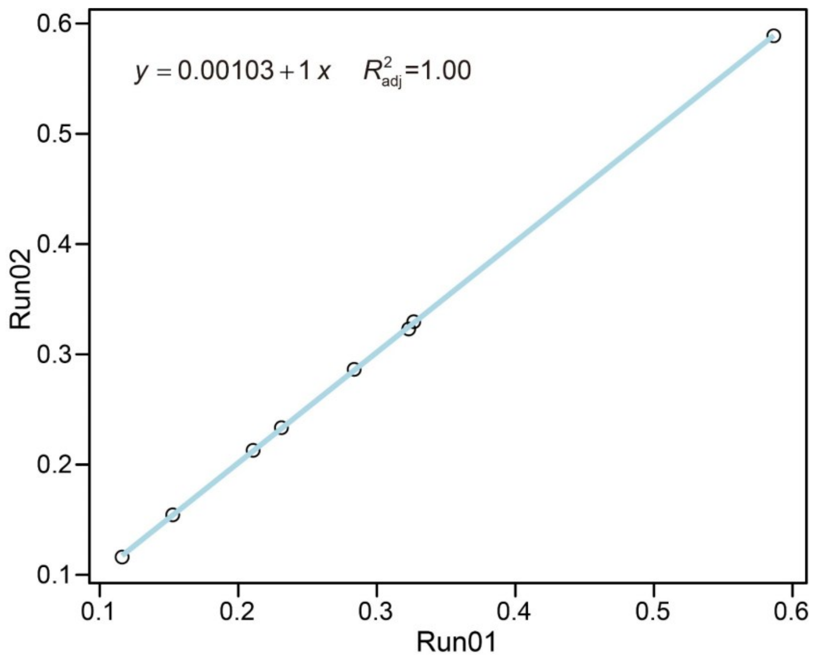

FIG S2 The convergence validation of the time tree. The parameters for two independent runs are: nsample is 400,000, burnin number is 160,000, and the sampfreq is 10.

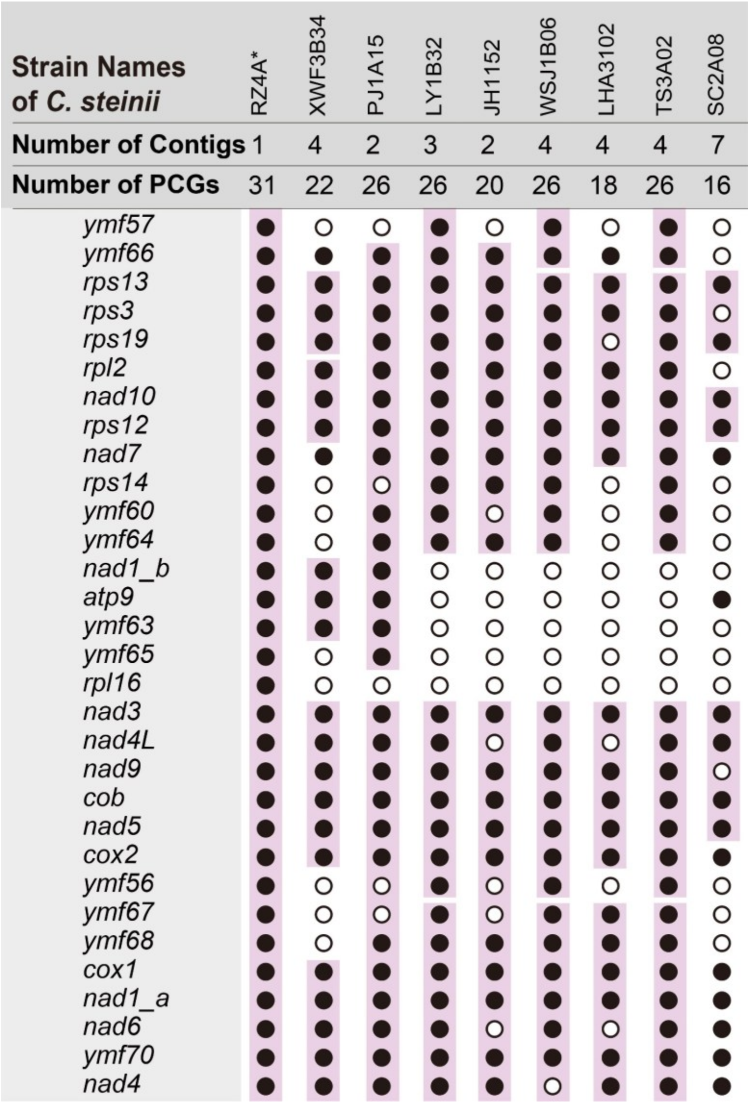

FIG S3 The highly conserved gene orders in mitogenomes of nine *C. steinii* strains.

* denotes the strain for *de novo* mitogenome assembly using long-read sequencing; hollow circles, unannotated protein-coding genes (PCGs); pink background indicates a continuous contig; solid circles, annotated PCGs in the *C. steinii* mitogenomes.

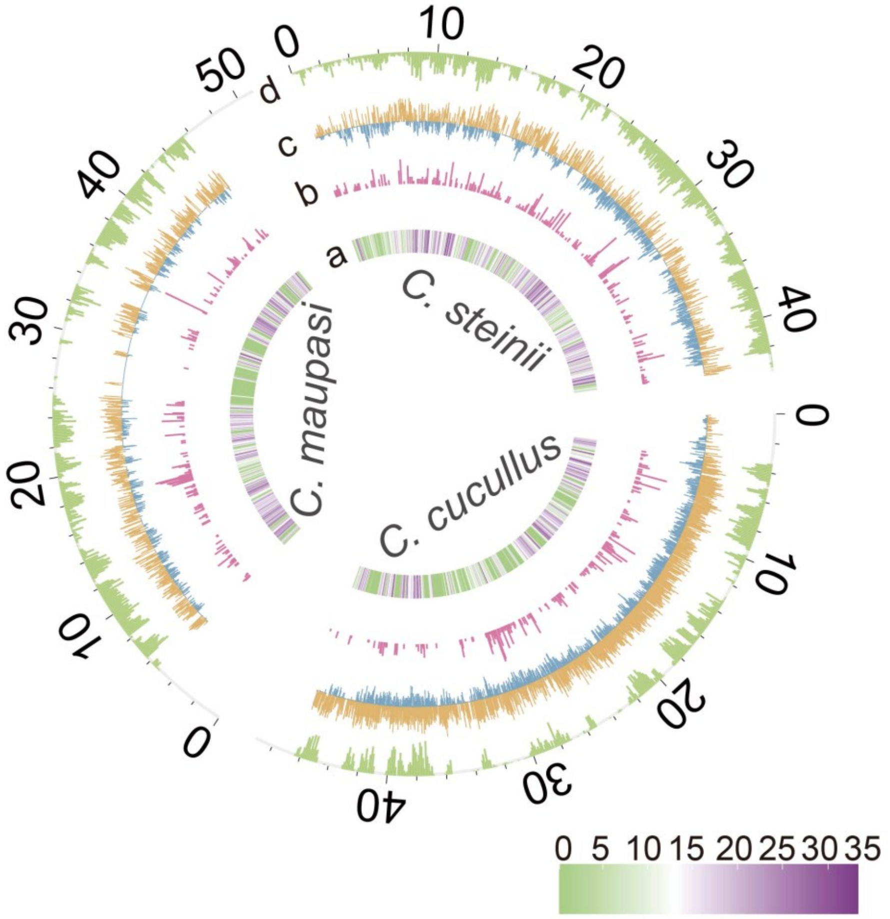

FIG S4 Population genetic parameters of three *Colpoda* mitogenomes. Track a shows density of SNPs (window size = 100 bp, step size =100 bp, non-overlapping). Track b represents indel density (window size = 100 bp, step size =100bp, non-overlapping). Lines in track c indicates variation of Tajima's D, where the values higher than zero are in orange, those less than zero are in blue. Track d shows the π values.

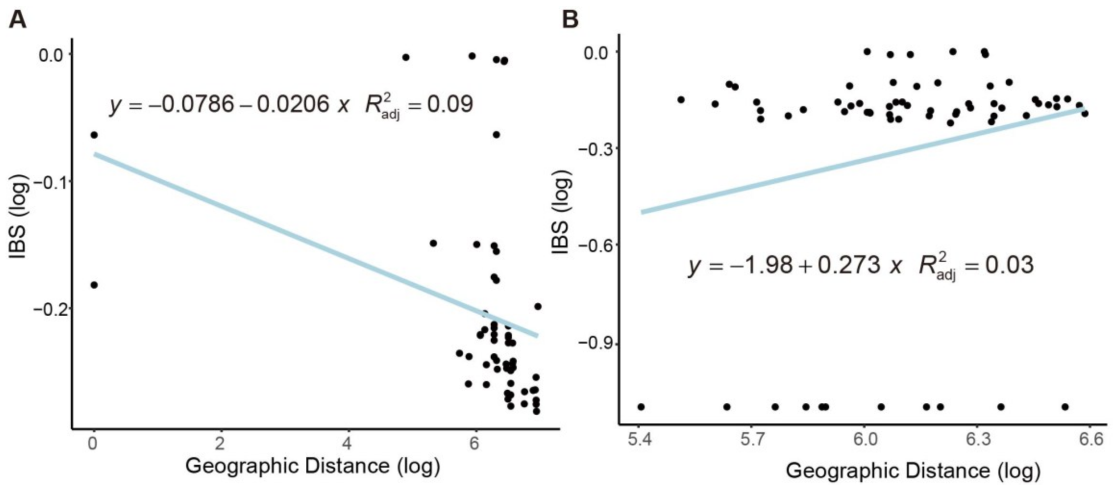

FIG S5 Scatter plot of IBS (Isolation by distance) versus geographic distances (in km), showing little correlation between geographic and genetic distance. A. *C. cucullus*, B. *C. steinii*.

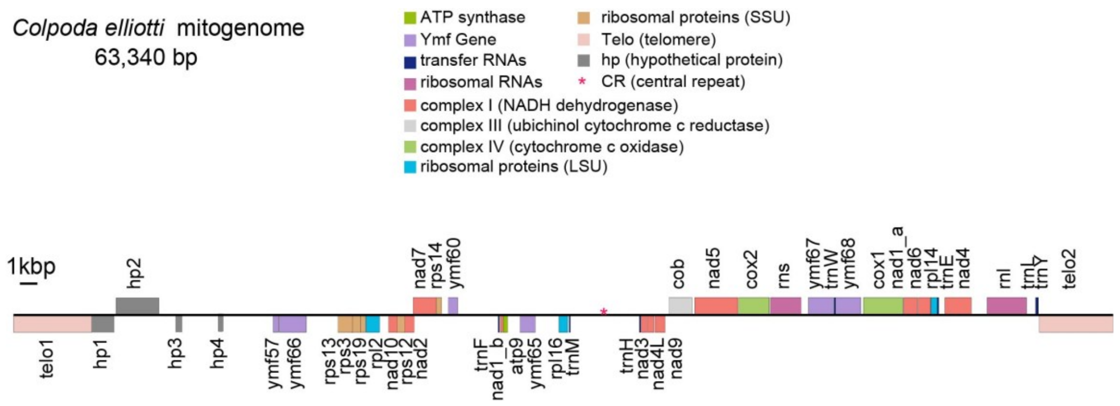

FIG S6 Genome atlas of *Colpoda elliotti* mitogenome. Rectangles in dark gray represent the hypothetical proteins.
