# Supplementary material for: Mitogenomic architecture and evolution of the soil ciliates Colpoda
Source: mSystems. 2024 Jan 23;9(2):e01161-23. doi: 10.1128/msystems.01161-23 (PMC10878089; doi:10.1128/msystems.01161-23)
Supplement: Supplemental text — Details of the materials and methods. [file msystems.01161-23-s0002.pdf]

Mitogenomic architecture and evolution of the soil ciliates *Colpoda*

Yuanyuan Zhang[1,2], Haichao Li[1], Yaohai Wang[1], Mu Nie[1], Kexin Zhang[1], Jiao Pan[1], Yu Zhang[1,3], Zhiqiang Ye[4], Rebecca A. Zufall[5], Michael Lynch[6], Hongan Long[1,2,*]

1. Key Laboratory of Evolution and Marine Biodiversity (Ministry of Education), Institute of Evolution and Marine Biodiversity, KLMME, Ocean University of China, Qingdao, Shandong Province, China 266003
2. Laboratory for Marine Biology and Biotechnology, Laoshan Laboratory, Qingdao, Shandong Province, China 266237
3. School of Mathematics Science, Ocean University of China, Qingdao, Shandong Province, China 266000
4. School of Life Sciences, Central China Normal University, Wuhan, Hubei Province, China 430079
5. Department of Biology and Biochemistry, University of Houston, Houston, Texas, USA 77204
6. Biodesign Center for Mechanisms of Evolution, Arizona State University, Tempe, Arizona, USA 85287

*Corresponding author: longhongan@ouc.edu.cn

SUPPLEMENTARY MATERIALS AND METHODS

Sample Collection, Species Identification and Culture

We collected topsoil samples across China (Fig. 1A, Table S1) and Italy (Rome, 12°26’35”E, 41°53’56”N). Subsequently, these soil samples were promptly transported back to our laboratory and preserved in a -80 °C freezer. Prior to the isolation of *Colpoda*, we followed the non-flooded petri dish method originally proposed by Foissner (1). After thawing and soaking the samples for a period of 48 hours, single *Colpoda* cells were isolated with an oral pipette under a dissection microscope. Each cell underwent a thorough cleaning process, which involved three consecutive rinses with 1× Penicillin-Streptomycin-Amphotericin B solution. The isolated *Colpoda* single cells were then cultured in wells of a 24-well plate (Nest, No. 702002). To initiate the cloning culture, we introduced 800 μL *E. coli* suspension with OD600 of 0.2. Following this incubation period, we picked 3–5 ciliates from each well, subjecting them to a heat-lysis process in preparation for the amplification of the 18S rDNA sequence. The PCR reaction system employed in this study consisted of 1 μL lysed ciliates, 7.5 μL 2× Phanta Flash Master Mix (Vazyme, No. P501-01), 0.5 μL forward primers (5'-GAAACTGCGAATGGCTC-3'), 0.5 μL reverse primers (5'-TACTGATATGCTTAAGTTCAGCGG-3') and 5.5 μL sterilized water. The PCR conditions were as follows: denaturation for 30 s at 98 °C, then 18 cycles for denaturation at 98 °C for 30 s, annealing for 30 s and extension at 72 °C for 30 s, during which the annealing temperature is 69–51 °C with the temperature dropping 1 °C per cycle and then another 18 cycles: denaturation at 98 °C for 30 s, annealing at 51 °C for 30 s and extension at 72 °C for 30 s, followed by the final extension for 5 min at 72 °C. To identify the species, we

searched the NCBI BLAST (https://www.ncbi.nlm.nih.gov/) database. Finally, the cultured ciliates were further expanded in quantity at a constant temperature of 25 °C and in *E. coli* suspension (final OD600 = 0.2).

Nucleic Acid Extraction, Library Construction and Sequencing

We harvested mass-cultured *Colpoda* cells by centrifuging at low speed (~600 g) to concentrate the ciliates, while minimizing bacterial contamination. For the extraction of total genomic DNA, we used the MasterPure™ Complete DNA and RNA Purification kit (Lucigen, No. 16339) and Mag-Bind® Blood&Tissue DNA HDQ 96 Kit (Omega, No. M6399). To prepare DNA libraries for sequencing, we employed the TruePrep® DNA Library Prep Kit V2 for Illumina Kit (Vazyme, No. TD501) and the TruePrep® Index Kit V2 for Illumina Kit (Vazyme, No. TD202) (2). Then, the concentration and quality of the DNA samples were assessed using the Qubit 3.0 (Thermo Fisher Scientific, US) and Nano-300 Micro-Spectrophotometer (ALLSHENG, China). For size selection of the sequencing libraries, we used the E.Z.N.A.®Gel Extraction Kit (OMEGA, No. D2500-02). Regarding the reference strains of the four *Colpoda* species, we generated HiFi reads using Circular Consensus Sequencing (CCS) through a PacBio Sequel II sequencer (Berry Genomics, Beijing, China). For the reference strains of the other two species (*C. inflata* and *C. steinii*), Nanopore reads were generated using a MinION sequencer in our laboratory. The sequencing of the remaining natural strains was performed on an Illumina HiSeq 4000 sequencer with PE150 sequencing, by Novogene (Beijing, China) and Berry Genomics (Beijing, China).

For RNAseq data, we employed the Single Cell Full Length mRNA Amplication Kit

(Vazyme, N712) and the TruePrep™ DNA Library Prep Kit V2 for Illumina® (Vazyme, TD503) to perform mRNA reverse transcription and construct RNA libraries specifically for verifying hypothetical proteins in *C. elliotti*, starting with 10–20 lysed cells as input. Then Illumina HiSeq 4000 sequencer was used to generate approximately 66Gbp of PE150 reads (Novogene, Beijing, China).

Mitogenome Assembly and Annotation

For the reference strains of four species (*C. cucullus* MLY2A28, *C. elliotti* LHA5931, *C. lucida* DZG2A41, *C. maupasi* MW2A28), we used pbccs (https://github.com/nlhepler/pbccs), extracthifi (v1.0.0, https://github.com/nlhepler/pbccs) and SAMtools v1.7 (3) to transform raw subreads to HiFi reads. To eliminate contamination from bacteria, specifically *Escherichia coli* K12 MG1655 (GenBank: GCA_000005845.2), we applied bwa mem v0.7.17-r1188 aligner (4) and SAMtools with the parameters "-b -S -f 4". For the other two reference strains (*C. inflata* RL4B, *C. steinii* RZ4A), we used NanoLyse v1.1.0 and NanoFilt v2.2.0 (5) to filter out sequences with poor quality and contamination. Draft genomes were assembled using canu v2.2 (6). Contigs with GC content exceeding 45% were filtered out using seqtk (v1.3-r106, https://github.com/lh3/seqtk). Mitochondrial contigs were identified by BLAST v2.11.0+ (7), against the published *Colpoda* mitochondrial genes (GenBank: FJ905159.1, EF070327.1). Additionally, we assembled the mitogenomes of *Colpoda* natural strains using unicycler v0.4.8 (8).

For mitogenome annotation, we used GeSeq (https://chlorobox.mpimp-golm.mpg.de/geseq.html), a web tool for organelle genome annotation. This tool was used to

annotate open reading frames (ORFs) longer than 150 nt, with reference to published mitogenomes of ciliates in the database. In addition, we also performed manual annotation using the following procedures: TRF (Tandem Repeat Finder) v4.10.0 (9) was used to identify telomeres and central repeats with parameters "2 7 7 80 10 50 500 -m -f -d". ORFs longer than 150 nt were predicted using ORFfinder (https://www.ncbi.nlm.nih.gov/orffinder/). blastx was used to search these ORFs against the mitogenome database (https://www.ncbi.nlm.nih.gov/genome/organelle/) to identify protein-coding genes (PCGs). rRNA and tRNA genes were detected using infernal v1.4.4 (10) and tRNA-scanSE (http://lowelab.ucsc.edu/tRNAscan-SE/) (11), respectively. Homologous genes were predicted using Orthofinder v2.5.4 (12). To visualize the genome structures, we used OGDraw (13) (https://chlorobox.mpimp-golm.mpg.de/OGDraw.html). Finally, for the analysis of stop codon usage in *Colpoda* mitogenomes, we used online tools from the Sequence Manipulation Suite (http://www.detaibio.com/sms2/codon_usage.html).

Mitogenome Collinearity and Phylogenomic Analysis

We used MUMmer v3.23 (14) to assess the genome collinearity of interspecific *Colpoda* mitogenomes. Specifically, we utilized the nucmer, delta-filter and show-coords modules to search for, filter and format collinear blocks. We set the maximum gap length between two adjacent matches (--maxgap) to 500 and the minimum matching length for clustering (--mincluster) to 100. To visualize the collinearity relationships, we utilized the advanced Circos function (15) of TBtools v1.068 (16).

To elucidate the phylogenomic relationships among *Colpoda* species, we concatenated 22

core PCGs in the mitogenomes of six *Colpoda* species, identified using Orthofinder. These core PCGs included *nad1* (split into two parts), *nad3*, *nad4*, *nad4L*, *nad6*, *nad10*, *rpl2*, *rpl16*, *rps12*, *rps13*, *rps14*, *rps19*, *cox1*, *cox2*, *cob*, *atp9*, *ymf57*, *ymf65*, *ymf66*, *ymf67* and *ymf68*. Three *Tetrahymena* species, *T. thermophila, T. pyriformis* and *T. paravorax* were included as outgroups. We used MUSCLE v3.8.31 (17), ParaAT v2.0 (18) and Gblocks v0.91b (19) for sequence alignment by codons and gap removal. The best-fit model, GTR+F+R3, was determined using the modelfinder program of IQ-TREE v2.0.3. RAxML-NG v.1.1(20) was used to construct the tree with 1000 bootstrap replicates.

In the absence of genomic and fossil evidence, we estimated the divergence time of *Colpoda*, by referring to the divergence time of Oligohymenophorea ciliates based on fossil records (21-23). We established approximate calibration time points, setting 116 Mya for the divergence of *T. thermophila* and *T. pyriformis*, and 323 Mya for *T. thermophila* and *T. paravorax*, according to the results in (23). The aligned mitochondrial genome sequences served as input data. We used the MCMCtree package of paml v4.9j (http://abacus.gene.ucl.ac.uk/software/paml.html) (24) to estimate divergence times with the following parameters: burnin = 40000, nasample = 200000, sampfreq = 10. To ensure the convergence of time trees, we independently ran two seeds and validated convergence according to the instruction manual of MCMCtree (http://abacus.gene.ucl.ac.uk/software/MCMCtree.Tutorials.pdf). The resulting tree was visualized using FigTree v1.4.4 (http://tree.bio.ed.ac.uk/software/Fig.tree/).

To explore divergence within and between *Colpoda* species, we utilized the mito-SSU rRNA gene as an indicator, following the rationale outlined by M. Dunthorn et al. (25). We

retrieved two mito-SSU rDNA sequences of *Tetrahymena* species (used as outgroups) from the NCBI Gene database. We then pooled them with another 33 newly sequenced mito-SSU rDNA sequences of *Colpoda* from this study for single-gene phylogenetic analysis. Alignments were done using MUSCLE v3.8.31 (17). A total of 1563 sites were used for constructing the single-gene tree, by RAxML-NG v.1.1 with the TVM+F+G4 model for 100 times.

Gene Rearrangement Analysis

To investigate interspecies gene rearrangement events in the evolutionary history of *Colpoda* mitogenomes, we used qMGR (26). This analysis involved comparing gene order with that of a reference mitogenome, specifically the mitogenome of *C. maupasi*. This process involves calculating a rearrangement score (RS) for individual genes within the mitogenomes. The RS for a single gene ($RS_{i,j}$) is determined by monitoring changes in the positions of the two nearest neighbor genes relative to that single gene. The initial RS for a single gene is set to 0, when there are no changes on either side of the gene. If there is a change on one side, the RS is assigned a value of 1. In cases where changes occur on both sides of the gene, the RS is 2.

A rearrangement frequency (*RFi*, a relative cumulative RS, %) for gene i can be defined as:

$$RFi = \frac{1}{2n}\sum_{j=1}^{n} RS_{i,j} * 100.$$

A rearrangement score (*RSj*) for species j can be defined as:

$$RSj = \sum_{i=1}^{k} RS_{i,j}.$$

In the case of analyzing natural isolates of the same species (*C. cucullus*, *C. maupasi*, and

C. steinii), the assembled mitogenome contigs were initially aligned with the corresponding reference mitogenomes. We used blastn to identify PCGs. Then, we examined the order and arrangement of these PCGs to investigate the intraspecies rearrangement patterns.

Selective Pressure Analysis

Ka/Ks, denoting the ratio between nonsynonymous and synonymous substitution rates, serves as a key estimator for assessing selective pressures acting upon the PCGs. A Ka/Ks value of 1 signifies neutral selection, suggesting no significant selective pressure on the gene. If Ka/Ks value exceeds 1, it indicates positive selection, or advantageous alleles are fixing in the population; conversely, Ka/Ks < 1 means that natural selection is removing deleterious mutations. It's important to note that this theory operates on the assumption that selection primarily targets protein-level changes, while mutations at the nucleic acid level tend to have minimal influence on amino acid sequences and are remain relatively unaffected by natural selection (27).

To estimate the selection pressures acting on *Colpoda* mitogenomes, we conducted alignments of nucleotide sequences at the codon level using MAFFT v7.505 (28) and ParaAT v2.0 (18). Subsequently, Ka/Ks ratios were calculated for 410 gene pairs of 35 orthogroups, using KaKs_Calculator v2.0 (29). We applied the MA model, a model that averages parameters across 14 candidate models. This analysis allowed us to assess the evolutionary rate of each coding gene. Then, we implemented a sliding window method to calculate the values of Ka, Ks and Ka/Ks values of each gene, to detect the selection pressure variation between highly diverse and relatively conserved genes, using Python scripts to extract intragenic slide-window

sequences, with a window size of 57 bp and a step size of 6 bp. These sequences were then subjected to calculations using KaKs_Calculator v2.0, with the 4-Mold Mitochondrial code chosen for the calculation.

Population Genetics Analysis

For the natural isolates of each species, sequencing was performed using an Illumina HiSeq4000 sequencer (PE150) (Novogene, Beijing, China; Berry Genomics, Beijing, China). To ensure data quality, sequencing adapters and low-quality reads were removed using fastp v0.20.1 (30)), with default parameters. Additionally, as previously mentioned, any traces of food bacteria contamination were eliminated. To align the clean reads to the reference mitogenome, we used BWA v0.7.17-r1188. Isolates with a total mitogenome coverage less than 20× were excluded from further analysis. Genome Analysis Toolkit (GATK) v4.1.2.0 (31) was used to call SNPs/Indels, and filtered by setting the following parameters: Depth < 2.0, Fisher Strand (FS) > 60.0, Mapping Quality (MQ) < 60.0, Mapping Quality Rank Sum (MQRankSum) < -12.5, or Read Position Rank Sum (ReadPosRankSum) < -8.0; Indels were filtered by Depth < 2.0, Fisher Strand (FS) > 200.0, or Mapping Quality Rank Sum (MQRankSum) < -20.0. SNP sites missed in over 20% of the samples were also excluded from further analysis. Vcftools v0.1.16 (32) was used to calculate density of SNPs. Candidate heteroplasmic sites were inferred based on the following criteria: a) Supported by a minimum of 3 reads; b) No potential Indels were identified within a 3-bp flanking sequence in each direction; c) Possess no more than 2 alternate alleles. Linkage disequilibrium (LD) decay was assessed using PopLDdecay v3.42 (33). Intra-species nucleotide diversity π was calculated by lab-developed python scripts. Plink

v1.9 (34) and R package geosphere (https://CRAN.R-project.org/package=geosphere) were used to calculate genetic distance IBS (identity by states) and geographical distance.

REFERENCE

1. Bardele CF, Blanton R. 2007. Morphology, morphogenesis and systematic position of the sorocarp forming ciliate *Sorogena stoianovitchae* Bradbury & Olive, 1980. *J Eukaryotic Microbiol* **38**:7-17.
2. Li H, Wu K, Ruan C, Pan J, Wang Y, Long H. 2019. Cost-reduction strategies in massive genomics experiments. *Mar Life Sci Technol* **1**:15-21.
3. Danecek P, Bonfield JK, Liddle J, Marshall J, Ohan V, Pollard MO, Whitwham A, Keane T, McCarthy SA, Davies RM, Li H. 2021. Twelve years of SAMtools and BCFtools. *Gigascience* **10**:giab008.
4. Li H. 2013. Aligning sequence reads, clone sequences and assembly contigs with BWA-MEM. *arXiv: Genomics* **14669139**.
5. De Coster W, Rademakers R. 2023. NanoPack2: population-scale evaluation of long-read sequencing data. *Bioinformatics* **39**:btad311.
6. Nurk S, Walenz BP, Rhie A, Vollger MR, Logsdon GA, Grothe R, Miga KH, Eichler EE, Phillippy AM, Koren S. 2020. HiCanu: accurate assembly of segmental duplications, satellites, and allelic variants from high-fidelity long reads. *Genome Res* **30**:1291-1305.
7. Altschul SF, Madden TL, Schäffer AA, Zhang J, Zhang Z, Miller W, Lipman DJ. 1997. Gapped BLAST and PSI-BLAST: a new generation of protein database search programs. *Nucleic Acids Res* **25**:3389-402.
8. Wick RR, Judd LM, Gorrie CL, Holt KE. 2017. Unicycler: Resolving bacterial genome assemblies from short and long sequencing reads. *PLoS Comput Biol* **13**:e1005595.
9. Benson G. 1999. Tandem repeats finder: a program to analyze DNA sequences. *Nucleic Acids Res* **27**:573-80.
10. Kalvari I, Nawrocki EP, Ontiveros-Palacios N, Argasinska J, Lamkiewicz K, Marz M, Griffiths-Jones S, Toffano-Nioche C, Gautheret D, Weinberg Z, Rivas E, Eddy SR, Finn RD, Bateman A, Petrov AI. 2021. Rfam 14: expanded coverage of metagenomic, viral and microRNA families. *Nucleic Acids Res* **49**:D192-D200.
11. Lowe T, Chan P. 2016. TRNAscan-SE On-line: integrating search and context for analysis of transfer RNA genes. *Nucleic Acids Res* **44**:gkw413.
12. Emms DM, Kelly S. 2019. OrthoFinder: phylogenetic orthology inference for comparative genomics. *Genome Biol* **20**:238.
13. Greiner S, Lehwark P, Bock R. 2019. OrganellarGenomeDRAW (OGDRAW) version 1.3.1: expanded toolkit for the graphical visualization of organellar genomes. *Nucleic Acids Res* **47**:W59-W64.

14. Kurtz S, Phillippy A, Delcher AL, Smoot M, Shumway M, Antonescu C, Salzberg SL. 2004. Versatile and open software for comparing large genomes. *Genome Biol* **5**:R12.
15. Chen C, Wu Y, Xia R. 2022. A painless way to customize Circos plot: from data preparation to visualization using TBtools. *iMeta* **1**:e35.
16. Chen C, Chen H, Zhang Y, Thomas HR, Frank MH, He Y, Xia R. 2020. TBtools: an integrative toolkit developed for interactive analyses of big biological data. *Mol Plant* **13**:1194-1202.
17. Edgar RC. 2004. MUSCLE: multiple sequence alignment with high accuracy and high throughput. *Nucleic Acids Res* **32**:1792-7.
18. Zhang Z, Xiao J, Wu J, Zhang H, Liu G, Wang X, Dai L. 2012. ParaAT: a parallel tool for constructing multiple protein-coding DNA alignments. *Biochem Biophys Res Commun* **419**:779-81.
19. Castresana J. 2000. Selection of conserved blocks from multiple alignments for their use in phylogenetic analysis. *Mol Biol Evol* **17**:540-52.
20. Kozlov AM, Darriba D, Flouri T, Morel B, Stamatakis A. 2019. RAxML-NG: a fast, scalable and user-friendly tool for maximum likelihood phylogenetic inference. *Bioinformatics* **35**:4453-4455.
21. Weitschat W, Guhl W. 1994. *Erster nachweis* fossiler ciliaten. *PalZ* **68**:17-31.
22. Jiang CQ, Wang GY, Xiong J, Yang WT, Sun ZY, Feng JM, Warren A, Miao W. 2019. Insights into the origin and evolution of Peritrichia (Oligohymenophorea, Ciliophora) based on analyses of morphology and phylogenomics. *Mol Phylogenet Evol* **132**:25-35.
23. Xiong J, Yang W, Chen K, Jiang C, Ma Y, Chai X, Yan G, Wang G, Yuan D, Liu Y, Bidwell SL, Zafar N, Hadjithomas M, Krishnakumar V, Coyne RS, Orias E, Miao W. 2019. Hidden genomic evolution in a morphospecies-the landscape of rapidly evolving genes in *Tetrahymena*. *PLoS Biol* **17**:e3000294.
24. Yang Z. 2007. PAML 4: phylogenetic analysis by maximum likelihood. *Mol Biol Evol* **24**:1586-91.
25. Dunthorn M, Foissner W, Katz LA. 2011. Expanding character sampling for ciliate phylogenetic inference using mitochondrial SSU-rDNA as a molecular marker. *Protist* **162**:85-99.
26. Zhang J, Kan X, Miao G, Hu S, Sun Q, Tian W. 2020. qMGR: a new approach for quantifying mitochondrial genome rearrangement. *Mitochondrion* **52**:20-23.
27. Hurst L. 2002. The Ka/Ks ratio: diagnosing the form of sequence evolution. *Trends Genet* **18**:486.
28. Rozewicki J, Li S, Amada KM, Standley DM, Katoh K. 2019. MAFFT-DASH: integrated protein sequence and structural alignment. *Nucleic Acids Res* **47**:W5-W10.
29. Wang D, Zhang Y, Zhang Z, Zhu J, Yu J. 2010. KaKs_Calculator 2.0: a toolkit incorporating gamma-series methods and sliding window strategies. *Genomics, Proteomics Bioinf* **8**:77-80.
30. Chen S, Zhou Y, Chen Y, Gu J. 2018. fastp: an ultra-fast all-in-one FASTQ preprocessor. *Bioinformatics* **34**:i884-i890.

31. Poplin R, Ruano-Rubio V, Depristo M, Fennell T, Carneiro M, Auwera G, Kling D, Gauthier L, Levy-Moonshine A, Roazen D, Shakir K, Thibault J, Chandran S, Whelan C, Lek M, Gabriel S, Daly M, Neale B, Macarthur D, Banks E. 2017. Scaling accurate genetic variant discovery to tens of thousands of samples. *bioRxiv* **201178**.
32. Myers S, Bottolo L, Freeman C, McVean G, Donnelly P. 2005. A fine-scale map of recombination rates and hotspots across the human genome. *Science* **310**:321-4.
33. Zhang C, Dong SS, Xu JY, He WM, Yang TL. 2019. PopLDdecay: a fast and effective tool for linkage disequilibrium decay analysis based on variant call format files. *Bioinformatics* **35**:1786-1788.
34. Purcell S, Neale B, Todd-Brown K, Thomas L, Ferreira MA, Bender D, Maller J, Sklar P, de Bakker PI, Daly MJ, Sham PC. 2007. PLINK: a tool set for whole-genome association and population-based linkage analyses. *Am J Hum Genet* **81**:559-75.
