# Supplementary material for: Mitogenomic architecture and evolution of the soil ciliates Colpoda
Source: mSystems. 2024 Jan 23;9(2):e01161-23. doi: 10.1128/msystems.01161-23 (PMC10878089; doi:10.1128/msystems.01161-23)
Supplement: Supplemental Tables — Tables S1 and S2. [file msystems.01161-23-s0003.pdf]

Mitogenomic architecture and evolution of the soil ciliates *Colpoda*

Yuanyuan Zhang[1,2], Haichao Li[1], Yaohai Wang[1], Mu Nie[1], Kexin Zhang[1], Jiao Pan[1], Yu Zhang[1,3], Zhiqiang Ye[4], Rebecca A. Zufall[5], Michael Lynch[6], Hongan Long[1,2,*]

1. Key Laboratory of Evolution and Marine Biodiversity (Ministry of Education), Institute of Evolution and Marine Biodiversity, KLMME, Ocean University of China, Qingdao, Shandong Province, China 266003
2. Laboratory for Marine Biology and Biotechnology, Laoshan Laboratory, Qingdao, Shandong Province, China 266237
3. School of Mathematics Science, Ocean University of China, Qingdao, Shandong Province, China 266000
4. School of Life Sciences, Central China Normal University, Wuhan, Hubei Province, China 430079
5. Department of Biology and Biochemistry, University of Houston, Houston, Texas, USA 77204
6. Biodesign Center for Mechanisms of Evolution, Arizona State University, Tempe, Arizona, USA 85287

*Corresponding author: longhongan@ouc.edu.cn

TABLE S1

TABLE S2

TABLE S1 Details of the newly-isolated *Colpoda* strains used in this study. Except for *C. cucullus* CLY0001, all other sampling cities are in China.

Species	Strain name	Sampling city	Longitude	Latitude	Sequencing depth	Breadth coverage	Clean reads (bp)	Sequencing platforms
C. cucullus	AR2C25	Baishan	127°47’46”E	42°16”8”N	72	74.39%	3,733,259,400	Illumina Novaseq6000 PE150
C. cucullus	WYJ002B	Changsha	112°57’37”E	28°10’5”N	73	76.58%	1,195,437,267	Illumina Novaseq6000 PE150
C. cucullus	CLY0001	Rome, Italy	12°26’35”E	41°53’56”N	38	52.91%	6,151,640,400	Illumina Novaseq6000 PE150
C. cucullus	JWY802A	Jingzhou	112°55’22”E	30°4’25”N	49	73.98%	4,936,097,100	Illumina Novaseq6000 PE150
C. cucullus	WK201A	Kaifeng	114°19’22”E	34°49’18”N	68	72.17%	1,230,294,444	Illumina Novaseq6000 PE150
C. cucullus	LWH0314	Kelamayi	84°51’49”E	44°19’22”N	74	81.81%	736,079,400	Illumina Novaseq6000 PE150
C. cucullus	CXY101A	Kuitun	84°30’5”E	44°58’40”N	123	87.53%	6,332,996,700	Illumina Novaseq6000 PE150
C. cucullus	MLY2A28	Sanya	109°30’0”E	18°15’0”N	137	100%	3,962,828,912	PacBio Sequel II CCS
C. cucullus	MLY2A29	Sanya	109°30’0”E	18°15’0”N	100	70.94%	3,892,762,754	Illumina Novaseq6000 PE150
C. cucullus	MLY2A30	Sanya	109°30’0”E	18°15’0”N	21	66.23%	2,731,327,200	Illumina Novaseq6000 PE150
C. cucullus	WYH502A	Tianshui	105°56’47”E	35°3’29”N	82	77.47%	7,479,183,300	Illumina Novaseq6000 PE150
C. elliotti	LHA5931	Gangtangcuo	86°41’56”E	32°17’13”N	220	100%	4,505,834,050	PacBio Sequel II CCS
C. inflata	RL4B	Qingdao	120°19’56”E	36°3’42”N	128	100%	19,230,000,000	Nanopore MinION R9.4
C. lucida	DZG2A41	Yichun	114°49’51”E	28°12’35”N	78	100%	6,161,771,312	PacBio Sequel II CCS
C. maupasi	MW2A28	Baiyin	105°3’8”E	35°41’27”N	94	100%	3,244,610,287	PacBio Sequel II CCS
C. maupasi	WYG102A	Beijing	116°16’40”E	40°2’49”N	73	82.99%	5,385,329,700	Illumina Novaseq6000 PE150
C. maupasi	KS101A	Foshan	113°16’21”E	22°44’33”N	68	79.20%	3,311,549,710	Illumina Novaseq6000 PE150
C. maupasi	ZGR701A	Haikou	110°24’27”E	20°1’3”N	60	75.57%	8,047,070,700	Illumina Novaseq6000 PE150

C. maupasi	QS402A	Kuerle	86°8’44”E	41°45’43”N	20	85.66%	5,727,276,300	Illumina Novaseq6000 PE150
C. maupasi	LT3A41	Shantou	116°34’50”E	23°16’19”N	37	98.14%	4,668,285,450	Illumina Novaseq6000 PE150
C. maupasi	WYH101B	Wenzhou	121°34’43”E	28°10’24”N	66	75.46%	2,628,027,600	Illumina Novaseq6000 PE150
C. maupasi	SC402A	Xi’an	108°46’4”E	32°2’19”N	36	79.08%	2,977,012,500	Illumina Novaseq6000 PE150
C. maupasi	SRZ0186	Xingtai	115°2’8”E	37°12’37”N	58	74.91%	9,602,548,800	Illumina Novaseq6000 PE150
C. maupasi	LMG1901	Zhoukou	114°45’20”E	33°22’50”N	57	74.91%	2,066,226,300	Illumina Novaseq6000 PE150
C. steinii	XWF3B34	Deyang	104°25’46”E	30°58’28”N	451	93.31%	30,618,057,600	Illumina Novaseq6000 PE150
C. steinii	TS3A02	Guiyang	106°36’17”E	26°35’35”N	1195	99.82%	35,171,925,600	Illumina Novaseq6000 PE150
C. steinii	PJ1A15	Hegang	130°14’38”E	47°16’35”N	2414	97.91%	27,137,886,000	Illumina Novaseq6000 PE150
C. steinii	WK1B14	Kaifeng	114°19’22”E	34°49’18”N	98	98.35%	482,663,100	Illumina Novaseq6000 PE150
C. steinii	LHA3102	Lasa	91°45’59”E	29°14’10”N	32	92.29%	12,167,609,100	Illumina Novaseq6000 PE150
C. steinii	RZ4A	Qingdao	120°19’56”E	36°3’42”N	80	100%	23,890,000,000	Nanopore MinION R9.4, Illumina Novaseq6000 PE150
C. steinii	MMY201A	Sanya	109°30’0”E	18°15’0”N	68	74.64%	3,204,197,400	Illumina Novaseq6000 PE150
C. steinii	SC2A08	Xi’an	108°46’4”E	34°2’19”N	271	54.13%	39,607,465,200	Illumina Novaseq6000 PE150
C. steinii	JH1152	Xianggelila	99°40’8”E	27°48’36”N	997	97.73%	26,289,569,700	Illumina Novaseq6000 PE150
C. steinii	DL201A	Xichang	102°15’29”E	27°53’24”N	42	81.91%	5,525,926,800	Illumina Novaseq6000 PE150
C. steinii	WSJ1B06	Xilinguole	116°3’27”E	43°53’52”N	1428	99.54%	42,191,794,500	Illumina Novaseq6000 PE150
C. steinii	LY1B32	Yushu	95°37’36”E	33°50’25”N	900	99.61%	32,738,315,700	Illumina Novaseq6000 PE150

TABLE S2 Length and GC content of four mitogenomes and their noncoding sequences.

Species	Mitogenome		Telomere			Central Repeat		
	Length (bp)	GC content (%)	Repeat unit	Length (bp)	GC Content (%)	Repeat unit	Length (bp)	GC Content (%)
C. cucullus	49189	21.04	ACACTCCCTTTCGGTCGTTAGAAAAGTTTATAC GCTCAGCTAACGCTTCGCTATTAGAAGTTTAA (65 bp)	3416	39.92	TATATAATAAT	231	0
C. elliotti	63340	20.22	ATTTATCGCTTCGCTATCATTTCACTTCGTTT (32 bp)	8745	34.39	TATATAAATATATTATATAT AAATATATTATATATATTA	1600	1.31
C. lucida	54804	21.37	CTCCCTGTCGGTCGTTATTAGTATGTTTAGCGT CACTTACGTTCCTATACGCTTACG (57 bp)	4045	42.15	AATTATAATTATTACTAATA ATAATAGAATTATAATAT	884	5.41
C. maupasi	51136	20.80	CATTGTTCTAAGAACTACGACGTTATATAAATT GCTGACGCAAATAATTCCACTATCGTTCTTACA ATTACATTGTTGATGAACTACGAAGCTAAGCTG CTGATAAAATCAGTCAAGCAGCGCAACCGCCC ATAATCAATTG (142 bp)	2373	21.78	AATTTATTTAAAAATTA	934	1.64
